# CHSY1 promotes CD8^+^ T cell exhaustion through activation of succinate metabolism pathway leading to colorectal cancer liver metastasis based on CRISPR/Cas9 screening

**DOI:** 10.1186/s13046-023-02803-0

**Published:** 2023-09-25

**Authors:** Guangshun Sun, Siqi Zhao, Zhongguo Fan, Yuliang Wang, Hanyuan Liu, Hengsong Cao, Guoqiang Sun, Tian Huang, Hongzhou Cai, Hong Pan, Dawei Rong, Yun Gao, Weiwei Tang

**Affiliations:** 1 Hepatobiliary/Liver Transplantation Center, the First Affiliated Hospital of Nanjing Medical University, Key Laboratory of Living Donor Transplantation, Chinese Academy of Medical Sciences, Nanjing, China; 2 Department of Breast Surgery, the First Affiliated Hospital With Nanjing Medical University, Nanjing, China; 3 Department of Surgery, the Second Affiliated Hospital of Jiaxing University, Jiaxing, Zhejiang China; 4 Department of Cardiology Zhongda Hospital, School of Medicine, Southeast University, Nanjing, China; 5 School of Basic Medicine, Nanjing Medical University, Nanjing, China; 6 Department of General Surgery, Nanjing First Hospital, Nanjing Medical University, Nanjing, China; 7 Department of Urology, Jiangsu Cancer Hospital &The Affiliated Cancer Hospital of Nanjing Medical University& Jiangsu Institute of Cancer Research, Nanjing, China

**Keywords:** CHSY1, Colorectal cancer, Immune escape, Liver metastasis, PD-L1

## Abstract

**Background:**

The most common site of metastasis in colorectal cancer (CRC) is the liver and liver metastases occur in more than 50% of patients during diagnosis or treatment. The occurrence of metastasis depends on a series of events known as the invasive-metastasis cascade. Currently, the underlying genes and pathways regulating metastasis initiation in the liver microenvironment are unknown.

**Methods:**

We performed systematic CRISPR/Cas9 screening using an in vivo mouse model of CRC liver metastasis to identify key regulators of CRC metastasis. We present the full results of this screen,which included a list of genes that promote or repress CRC liver colonization. By silencing these genes individually, we found that chondroitin sulfate synthase 1 (CHSY1) may be involved in CRC metastasis. We verified the function of CHSY1 and its involvement in liver metastasis of CRC through in vivo and in vitro experiments.

**Result:**

The results of TCGA and CRISPR/Cas9 showed that CHSY1 was overexpressed in CRC primary and liver metastasis tissues and indicated a worse clinical prognosis. In vitro and in vivo experiments confirmed that CHSY1 facilitated the liver metastasis of CRC and CHSY1 induced CD8^+^ T cell exhaustion and upregulated PD-L1 expression. The metabolomic analysis indicated that CHSY1 promoted CD8^+^ T cell exhaustion by activating the succinate metabolism pathway leading to liver metastasis of CRC. Artemisinin as a CHSY1 inhibitor reduced liver metastasis and enhanced the effect of anti-PD1 in CRC. PLGA-loaded Artemisinin and ICG probe reduced liver metastasis and increased the efficiency of anti-PD1 treatment in CRC.

**Conclusion:**

CHSY1 could promote CD8^+^ T cell exhaustion through activation of the succinate metabolic and PI3K/AKT/HIF1A pathway, leading to CRC liver metastasis. The combination of CHSY1 knockdown and anti-PD1 contributes to synergistic resistance to CRC liver metastasis. Artemisinin significantly inhibits CHSY1 activity and in combination with anti-PD1 could synergistically treat CRC liver metastases. This study provides new targets and specific strategies for the treatment of CRC liver metastases, bringing new hope and benefits to patients.

**Supplementary Information:**

The online version contains supplementary material available at 10.1186/s13046-023-02803-0.

## Background

One of the leading causes of cancer-related deaths is metastatic disease [1]. Metastasis is a complex multi-step process in the biological behavior of tumor cells, which involves many factors, such as the ability of cancer cells to spread and invade, the entry of disseminated tumor cells (DTCs) into the circulatory system, and the entry of DTCs from the circulatory system into distant organs. After a series of behaviors, it eventually develops into metastatic lesions, which are detected by clinical means. A complex network of intracellular and intercellular signal transduction cascades regulates these parts [2, 3]. An important feature of cancer metastasis is Organ-tropism, and many cancers exhibiting a metastatic preference for specific organs [4]. In spite of the “seed and soil” theory has been around for more than 100 years [5], the mechanisms of DTCs adapt and colonize new organ environments,and the key steps in the metastatic process, remain largely undefined.

Colorectal cancer (CRC) has risen to the second leading cause of cancer death worldwide, and like most solid tumors, metastasis progression is one of the important causes of CRC death [3, 6]. With the increase and improvement of clinical treatment methods, the long-term survival of metastatic CRC patients has been improved to a certain extent, but the five-year survival rate is still only a low 15% [7]. Liver is the most frequent site of CRC metastasis, an estimated 50% of patients develop liver metastases during diagnosis and treatment [3]. The occurrence of CRC liver metastases will go through a series of complex cascade reactions, which we call the invasion-metastasis cascade [8]. However, the detailed genes and specific mechanisms that regulate CRC liver metastasis are still inconclusive.

In bacteria, the CRISPR/Cas9 system is an RNA-based adaptive immune system. Various studies have suggested that intervening in the CRISPR/Cas9 system could affect many human genes to treat a variety of human diseases, including cancer. Not only that, but CRISPR/Cas9 gene editing is one of the most effective genomic techniques available. Other studies suggest that, in addition to being a potential cancer idea, the CRISPR/Cas9 technology could be used to improve the effectiveness of existing therapies [9, 10]. In the present study, we performed systematic CRISPR/Cas9 screening using an in vivo mouse model of CRC liver metastasis to identify key regulators of CRC metastasis.We revealed the complete data for this screen, listing the genes involved in promoting or inhibiting CRC liver metastases. Although some of these genes are involved in tumorigenesis, their role in metastatic colonization has not been well described. By silencing these genes individually, we found that chondroitin sulfate synthase 1 (CHSY1) may be involved in CRC metastasis. Our study found that CHSY1 can activate succinic acid metabolism pathway and promote CD8^+^ T cell failure, which causes liver metastasis of CRC. Meanwhile, the combination of CHSY1 knockdown with anti-PD1 may provide a new method for CRC immunotherapy. For further clinical translation, we found that Artemisinin significantly inhibited CHSY1 activity and that the combination with anti-PD1 could synergistically treat CRC liver metastases. Polylactic acid-glycolic acid (PLGA)-loaded Artemisinin-linked Indocyanine green (ICG) probes could further improve the therapeutic efficacy of Artemisinin and visualize drug metabolism in vivo.

## Materials and methods

### CRISPR/Cas9 sequencing and data analysis

The CRISPR Screen II sequencing library was constructed as follows. A two-step PCR method was used to construct the library, and different reaction numbers were determined according to the sample types. General primers are designed before and after the sgRNA sequence in the region, and the reaction system was constructed with high-fidelity enzymes. In the first step, PCR was used to obtain the target fragment, and the number of cycles was generally controlled within 18–22 cycles. In the second step, PCR was performed by adding primers (Index) and adapters (Adapter) to obtain the target fragments, and the number of cycles was generally controlled within 8–12 cycles. After the two-step PCR is completed, the glue is cut and cycled to obtain a target band. After library purification, qubit 3.0 (Invitrogen, Carlsbad, CA, USA) was used to quantify the library concentration and 2200 (AgilentTechnologies, Palo Alto, USA) was used to detect the library fragment size. Nano QC (Illumina, San Diego, USA) was arranged. DNA libraries with different index markers were mixed and 2 × 150 bp dual-end sequencing (PE) was performed according to the instrument instructions of Illumina (Illumina, San Diego,USA). HiSeq Control Software (HCS) + OLB + Gapeline-1.6 (Illumina) of the HiSeq instrument was used for image analysis and base detection. Finally, the raw sequencing data were analyzed by GENEWIZ bioinformatics analysts.

Target sgRNAs (19 bp-21 bp) were extracted using an in-house script based on the upstream and downstream 5 bp bases, and the extracted sgRNAs were quality controlled to obtain clean reads. The clean reads were compared with the sgRNA library using MAGeCK software and the results were counted. The results of differential gene analysis and GSEA enrichment analysis were compared using MAGeCK software.

### Single cell RNA sequencing analysis

We prepared stock solutions with DMEM, HEPES (Thermo Fisher, USA), high glucose, 1% bovine serum albumin (BSA; Thermo Fisher, USA), and GlutaMAX™ supplement to store the samples we collected. The samples included primary CRC tissue and corresponding normal intestinal tissue after surgical resection, as well as liver metastatic cancer tissue and corresponding normal liver tissue. After starting the experiment, cut the sample into small pieces of 0.5–1.0mm^2^ with a sterile scalpel, and then digest the sample. The Unique Molecular Identifier (UMI) was controlled between 3,000 and 40,000, and mitochondria were no more than 10% of the total UMI for raw counts. The single cells were then filtered for analysis. Finally, the characteristics of the single cell sequencing data of each group were analyzed in detail.

### Patient and tissue specimen collection

According to the Declaration of Helsinki, we have informed all participants of the process and purpose of the study and obtained written informed consent from all patients before the study began. The whole process of collecting human specimens has been approved by the Medical Ethics Committee of Nanjing Medical University in advance. In this experiment, primary colorectal cancer and corresponding intestinal tissue, liver metastatic cancer tissue and corresponding normal liver tissue, blood before surgery were collected from 1 patient with primary colorectal cancer for single-cell RNA sequencing. Primary colorectal cancer and corresponding intestinal tissue, liver metastatic cancer tissue and corresponding normal liver tissue from 4 patients with primary colorectal cancer for immunofluorescence. In addition, liquid nitrogen samples of CRC and paracancer tissues from 54 patients admitted to the Jiangsu Provincial People's Hospital and Nanjing Hospital affiliated to Nanjing Medical University were collected. We snap-frozen them in liquid nitrogen to protect the samples until the next use. The collected tumor specimens were analyzed and classified by experienced clinicians. We regularly followed up 54 CRC patients and summarized their relevant data, including tumor stage, tumor differentiation, tumor size, tumor lymph node metastasis stage, age, and gender. We defined overall survival in these 54 patients as the date of first surgery to the date of last follow-up or death.

### RNA extraction and qRT-PCR

To test the knockdown efficiency of the lentivirus we constructed against CHSY1 in human colorectal cancer cell lines HCT116, LOVO and mouse colorectal cancer cell line MC38, we performed qRT-PCR. Following the manufacturer's protocol, we used TRIzol reagent (Invitrogen, USA) to separate the total RNA from cells above. We reverse transcribed the RNA to cDNA using a reverse transcription kit (Takara, Japan). In addition, qRT-PCR was also used to detect PD-L1 expression in HCT116 and LOVO cells after CHSY1 knockdown. Table S1 lists all primer sequences. mRNA expression levels were normalized with an internal control (GAPDH).

### Cell and cell culture

Through the Cell Bank of Type Culture Collection (Chinese Academy of Sciences, China), we obtained human CRC cell lines and mouse CRC cell lines, including HCT116, LOVO and MC38. The obtained cells were cultured in RPMI 1640 medium (BI, USA) supplemented with 10% fetal bovine serum (FBS; Gibco, USA). A constant temperature incubator at 37 °C and 5% CO2 was used for cell culture.

### Cell transfection

We constructed plasmids and lentiviral packaging (Genechem, China) of CHSY1 in human and mouse CRC cells (HCT116, LOVO and MC38). By Human/Mouse shRNA (Genechem, China), CHSY1 was downregulated in both human CRC cells (HCT116 and LOVO) and mouse CRC cells (MC38). We cultured 1 × 10^5^ cells per well in a six-well plate and 2 ml of medium in an incubator for 24 h. The medium was then changed to 1 ml, and the appropriate amount of virus and 40 µL of polybrene (Sigma-Aldrich, USA) were added to it. After 12–16 h of incubation, the cells were cultured with normal medium and screened with puromycin. The sequences of the shRNAs constructed by the items are shown in Table S1. To observe the transfection efficiency, we performed qRT-PCR and Western blot experiments. Finally, Human-sh3-CHSY1 and Mouse-sh1-CHSY1 were used for further experiments. To perform overexpression experiments, we subcloned the cDNA of CHSY1 into pcDNA3.1. The cells were plasmid transfected using Lipofectamine 2000 (Invitrogen,USA). The plasmid vector pcDNA3.1 was used as a control transfection agent.

### Cell proliferation assay

LOVO cells and HCT116 cells were divided into experimental group and control group, and were seeded in 96-well plates respectively. 1000 cells were seeded in 100 μL of medium per well and treated with 10 μL of CCK-8 solution (RiboBio, China). At 0, 24, 48, and 72 h of culture, cell absorbance at 450 nm was measured using a microplate reading element. The measurement procedure followed manufacturer's instructions (Synergy, USA).

We performed Cell-Light 5-ethynyl-2'-deoxyuridine (EdU) experiments to evaluate the proliferation capacity of cells using the EdU DNA Cell Proliferation Kit (RiboBio, China). A 24-well plate was used to seed 50,000 cells per well. One day after normal culture, cells were incubated with 50 mmol/L EdU solution for 2 h, then fixed with 4% paraformaldehyde. Following the manufacturer's protocol, we treated the cell lines with Apollo Dye Solution and DAPI, respectively, and finally captured and counted under an Olympus FSX100 microscope (Olympus, Japan).

### Transwell migration and invasion assays

Following the manufacturer's instructions, we added 200 μl of serum-free RPMI 1640 medium to the upper chamber and seeded 20,000 HCT116 cells and LOVO cells, respectively, and divided them into experimental and control groups. To assess cell invasive capacity, we plated Transwell chambers (Corning, USA) with Matrigel mix (BD Biosciences, USA). The Matrigel mix was not used when assessing cell migratory capacity. 700 μl of RPMI 1640 medium containing 10% FBS was placed in the lower chamber to chelate the CRC cell chemotactic agent. After culturing in a normal incubator for 24 h, the upper chamber was removed, the serum-free medium was aspirated, fixed with 4% paraformaldehyde for 10 min, then stained with crystal violet (Kaigen, China) for 15 min, and finally washed with PBS. The cell lines were microscopically photographed and 5 areas were taken for counts.

### Wound healing assay

HCT116 and LOVO cells were seeded on 6-well culture plates to achieve a post-transfection process. Manual linear wound elimination was performed on the fused cell monolayer using a standard 20 μl pipette tip. After washing off the floating cells with PBS, add complete medium and incubate in a 37 °C incubator. Photographs were taken with an inverted microscope at 0 h, 24 h, and 48 h, and the scratch width was measured. Each group of experiments was performed in triplicate.

### Mass cytometry

The tumor tissues of two groups of mice (sh-NC group, sh-CHSY1 group) were harvested and treated with Miltenyi Mouse Tumor Dissociation Kit (Miltenyi Biotec, Germany) to obtain a certain amount of tumor cells for CyTOF staining. This was followed by FlowJo preprocessing and bioinformatics analysis. The experiment was performed at PLTTECH (Plttech, China).

### Immunofluorescence and immunohistochemistry assays

For immunofluorescence experiments, we cut the paraffin-embedded sample (including primary colorectal cancer and corresponding intestinal tissue, liver metastatic cancer tissue and corresponding normal liver tissue from patients with colorectal cancer liver metastases that we collected, and tumor tissues in subsequent mouse experiments.) into 4 mm thick. Cells were fixed with 4% formaldehyde for 20 min at room temperature. Permeabilize with PBS containing 0.05% Triton X-100 (Sigma-Aldrich, USA) for 5 min. Samples were sealed in PBS containing 2% BSA for one hour. Antibody-specific CHSY1 and PD-L1 (Abcam, UK) was incubated overnight at 4 °C, followed by Alexa fluorine-HRP conjugated secondary antibodies (Abcam, UK) for one hour at room temperature. DAPI (Sigma-Aldrich, USA) was used for Nuclei were reverse stained and photographed at the end. For immunohistochemistry experiments, we first incubated the samples (including samples of CRC liver metastases from mice in different treatment groups.) at 4 °C overnight, followed by the addition of 3'-diaminobenzidine, and CD8, CD4, PD1, PD-L1 and KI67 specific antibodies. After obtaining immunohistochemical and immunofluorescence images, imageJ software was used to calculate the proportion of positive regions and used for subsequent statistical analysis.

### SDH concentration detection

Succinate dehydrogenase concentrations were assessed colorimetrically using a commercially available Succinate Dehydrogenase assay kit (A022-1–1, Nanjing Jiancheng, China) and HCT116 cells and LOVO cells with knockdown or overexpression of CHSY1 were extracted according to the manufacturer's instructions. Samples were analyzed by loading onto 12-well plates and measuring absorbance at 600 nm.

### Western blotting method

Proteins were extracted from cells with RIPA buffer solution (Sigma-Aldrich, USA), dissolved on SDS–polyacrylamide gel, and then transferred to PVDF membrane. Primary antibodies CHSY1, P-PI3K, PI3K, AKT, PD-L1, P-AKT and HIF1A were used (Abcam, UK). Using peroxidase-bound secondary antibodies (Sigma-Aldrich, USA), chemiluminescence (ECL; Thermo Fisher, USA).

### Metabolomics sequencing and detection

Sh-CHSY1 LOVO cells and sh-NC LOVO cells were used for non-targeted metabolomics analysis, each group contains six samples. The protein was precipitated with methanol/acetonitrile (1:1, v/v), and the supernatant containing the same amount of cells in each sample was dried in a high-speed vacuum enrichment centrifuge. During the mass spectrometry test, 100 μL acetonitrile-aqueous solution (1:1, v/v) was added to redissolve, and centrifuged at 4℃ for 16000 g for 20 min. The supernatant was obtained for LC–MS/MS analysis. The chromatographic separation was performed by SHIMADZU-LC30 ultra-high performance liquid chromatography (UHPLC) using HILIC columns, followed by positive ion ( +) and negative ion (-) mode detection by electrospray ionization (ESI) for each sample. The samples were separated by UPLC and then analyzed by mass spectrometry using QE Plus mass spectrometer (Thermo Scientific) and ionized using HESI source. After obtaining the original data, MSDIAL software was used for peak alignment, retention time correction and peak area extraction. The metabolite structure was identified by precise mass number matching (mass tolerance < 20 ppm) and second-order spectral matching (mass tolerance < 0.02 Da) method to search HMDB, MassBank and other public databases and our self-built metabolite standard database. For the extracted data, delete the missing values in the group. 50% of ion peaks did not participate in subsequent statistical analysis; The total peak area of positive and negative ion data was normalized respectively, and the peaks of positive and negative ions were integrated and pattern recognition was carried out by R software. After the data were preprocessed by Unit variance scalin (UV), subsequent data analysis was conducted.

### Metabolome sequencing analysis

Raw MS data were processed using MS-DIAL. Using MS/MS data, public databases were compared to our own standard library of metabolites. All evaluation models were fitted by permutation tests. OPLS-DA allows the use of projected variable importance (VIP) analysis to identify metabolites. Mean VIPs greater than 1 were considered significant, and higher scores indicated more significant recognition, so this was the criterion for selecting biomarkers. At the level of univariate analysis, statistical significance thresholds and two-tailed Student's t-tests (*P*-values) were used to evaluate metabolites. In multiple group analyses, p-values were calculated using one-way analysis of variance (ANOVA). VIP values greater than 1.0 and P values less than 0.05 were considered statistically significant. The identified differential metabolites were analyzed by cluster analysis using the R package. The KEGG database (http://www.kegg.jp) performs KEGG pathway analysis on differential metabolite data. KEGG enrichment analysis was performed by multiple tests using Fisher's exact test and FDR correction. Enriched KEGG pathways were considered statistically significant at P values less than 0.05.

### Molecular docking

Molecular docking is used to verify the binding activity between an active ingredient and a key target. AutoDock Vina (Vina, version 1.1.2), a program that operates with a semi-flexible docking method with a docking accuracy of up to 78%, was used as a molecular docking program in this study. Download CHSY1 protein with ID Q86X52 from Uniprot database. The structure of the small molecule artemisinin was downloaded from the Pubchem database for docking. The CHSY1 protein was docked with the small molecule artemisinin. The small molecule artemisinin was designated as a ligand and the CHSY1 protein as a receptor. PyMOL (4.3.0 edition) software (https://pymol.org/) is used to separate the original ligand and protein structure, dehydration and removal of organic matter, AutodockTools (http://mgltools.scripps.edu/downloads) for hydrogenation, Check the charge, specify the atomic type as AD4 type, calculate the gasteiger, and construct the docking grid box of the protein structure, in addition, the chemical composition (small molecule ligand) should determine the Root, select the torsion bond of the ligand in AutodockTools. Finally, in AutodockTools, both the protein structure and the format of the small molecule ligand should be converted from ".pdb "to".pdbqt "for further docking. After Vina docking, the scores of the docking combination of CHSY1 protein and small molecule artemisinin were calculated, and the force analysis and visualization from three and two dimensions were carried out using Pymol and Discovery Studio software.

### Construction of PLGA-loaded Artemisinin ICG probe

In order to construct Artemisinin ICG probe, we first added 20 mg polyPLGA and 20ul artemisinin to 0.5 ml chloroform under ultrasound until completely dissolved. Then 50ul fluorescent aqueous solution was added into the solution, and the cell grinder ultrasonic was carried out at a certain power for 5 min, and then 3 ml 3% PVA solution was added to continue the ultrasonic mixing for 5 min. Finally, the resulting emulsion was added to 0.2% PVA solution and mechanically stirred for 8 h. After ultrafiltration cleaning and filtration at 4℃ through a 0.22um filter membrane. In order to load the obtained artemisinin ICG probe with PLGA, 10 mg PLGA microspheres were added to 100 mM PBS buffer solution, and 5 mg EDC and 2 mg fluorescence agent were added and stirred overnight. The obtained solution was ultrafiltered and filtered through 0.22um membrane to obtain the PLGA-loaded artemisinin ICG probe. Finally, the artemisinin ICG probes loaded with plga were stored in a refrigerator at 4℃ for later use.

### Animal models

The Animal Care Committee of Nanjing Medical University approved our animal experiments. All operations complied with the corresponding ethical standards of animal experimental institutions. All mice used in our experiments were housed under SPF conditions in the Experimental Animal Center of Nanjing Medical University. Mice were sacrificed by cervical dislocation.

To establish a mouse model of CRC liver metastasis, we injected 2 × 10^6^ MC38 cells (include sh-NC and sh-CHSY1) into the spleen of male C57BL/6 mice, respectively. The specific procedure is to first anesthetize the mice with an intraperitoneal injection of 0.5% sodium pentobarbital (50 mg/kg) and weigh them. After the mice were anesthetized, they were fixed on the operating table with tape. After disinfecting the skin, a longitudinal incision (approximately 0.5 cm) was made in the left axillary line of the mouse under the posterior border of the rib cage. A willow spleen was found and removed from the abdomen. Immediately after, a single-cell suspension (2 × 10^6^ MC38 cells) was injected into the lower pole of the spleen, and the injection site was wiped with an alcohol swab. Abdominal muscles and skin were sutured intermittently after no bleeding was found. After surgery, the mice were subjected to SPF conditions in an animal laboratory. Grouped as follows: sh-NC, sh-CHSY1, sh-NC + anti-PD1 (BP0273; Bioxcell,USA), and sh-CHSY1 + anti-PD1(*n* = 5 in each group). In the sh-CHSY1 + anti-PD1 group, 6.6 mg/kg anti-PD1 was injected intraperitoneally on day, and once every 4 days thereafter. On day 20, mice were sacrificed and their liver protein expression was examined by immunohistochemical analysis.

### GSE analysis, TCGA data analysis and site prediction

The tcgportal (http://tumorsurvival.org/index.html) was applied to evaluate the association of CHSY1 expression with the prognosis of CRC patients. We used hTFtarget database (http://bioinfo.life.hust.edu.cn/hTFtarget#!/) to analyze the transcriptional binding sites of PD-L1 and HIF1A. JASPAR database (http://jaspar.genereg.net/) was applied to seek the HIF1A binding sites in the PD-L1 gene promoter. We acquired ChIP data from GSE85096 and GSE85096 to seek human PD-L1 and HIF1A binding sites. The “Peak Browser” function of ChIP-Atlas was used to obtain ChIP-Atlas peak-call data for transcription factors and visualize protein binding at specific genomic loci with the IGV Genome Browser. Correlations between CHSY1 expression and immune factors were predicted using the TISIDB database (http://cis.hku.hk/TISIDB/index.php).

### Statistical analysis

GraphPad Prism 9.0 software served for statistical analysis. All data are in the form of mean ± SD(Standard Deviation). The 2 tail-student *t* test was used to compare 2 independent samples. ANOVA was used to determine variation between or between groups. Kaplan–Meier Plotter was applied for prognostic analysis. A *P* < 0.05 reported statistical significance.

## Results

### Results of CRISPR/Cas9 screening of CRC liver metastasis model in vivo

**Fig. 1:**
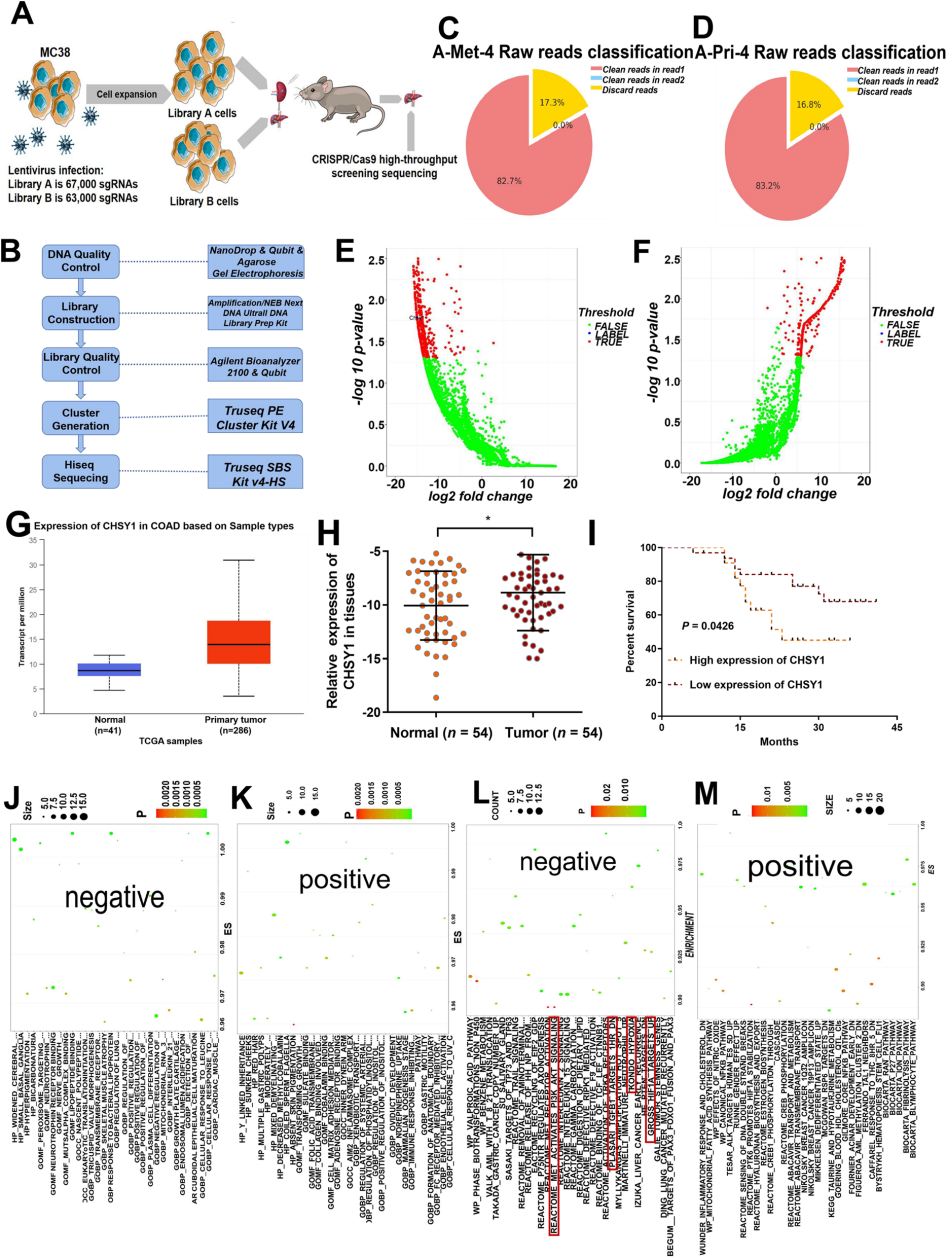
CRISPR/Cas9 screen results of CRC liver metastasis model in vivo. **A** Flow chart of CRISPR/Cas9 high-throughput screening sequencing**. B** The complete process of CRISPR/Cas9 screen technology. **C**-**D** Sequence composition of metastatic (**C**) and primary (**D**) foci. **E**–**F** Volcano plot of genes with significantly different negative(**E**) and positive (**F**) screening results. **G** TCGA results showed that CHSY1 was significantly overexpressed in CRC tissues compared with that in the corresponding paracancerous tissues. **H** The expression of CHSY1 was validated in qRT-PCR results of 54 CRC patients. **I** The CRC patients with high CHSY1 expression had reduced overall survival. **J**-**K** GO analysis pathway in negative (**J**) and positive (**K**) groups.(L-M) KEGG analysis pathway in negative (**L**) and positive (**M**) groups. *, *P* < 0.05

After genome-wide library screening of mouse colon cancer cell MC38 (including 130000sgRNA), we amplified the infected cells, and then used the amplified MC38 cells to construct a mouse liver metastasis model of colorectal cancer. Obtained mouse liver metastases were analyzed by CRISPR/Cas9 screening for high-throughput sequencing to identify key regulatory factors of colorectal cancer liver metastasis (Fig. 1A). Using CRISPR/Cas9 high-throughput screening technology for positive or negative screening in tumor cells, candidate gene-directed RNA (gRNA) can be enriched to search for target genes of interest. The complete process of this technology includes the following steps: lentiviral library construction, virus infection of cells within the library, experimental screening of cells, genomic DNA extraction and second-generation sequencing library construction, second generation sequencing and bioinformatics analysis (Fig. 1B). After obtaining raw data with sufficient sequencing depth, bioinformatic analysis was performed, which consists of the following three main stages. The detailed analysis process was shown in Figure S1A. According to the library construction method, a 20 bp gRNA sequence is extracted in an in-house custom script to obtain gRNA clean data for subsequent analysis. The composition of the raw sequenced sequences was counted according to the distribution of gRNA in the sequencing data, as shown in Fig. 1C-D. Figure S1B-C showed the average base mass values and the average sequencing error rate for each sample read at different sequencing positions, and the distribution statistics of base content were used to detect the separation phenomenon of AT and GC (Figure S1D-E). We used volcano plots to show genes with significant differences in negative and positive screening results (Fig. 1E-F). GO analysis revealed that the negative group was mainly enriched in lipopeptide binding, response to bacterial lipoproteins, peroxisomal target sequence binding, nerve growth factor receptor binding, cellular response to leucine and negative regulation of collagen metabolic processes, while the positive group was mainly enriched in multiple gastric polyps, norepinephrine uptake, fc receptor-mediated inhibitory signaling pathways and cobalamin binding pathways (Fig. 1J-K). KEGG analysis indicated that the negative group was mainly enriched in valproic acid pathway, reactome met activation of pi3k akt signaling, benzene metabolism, trail signaling and activation of trka receptors, while the positive group was mainly enriched in inflammatory response, mitochondrial fatty acid synthesis pathway, reactome sensing dna double-strand breaks, reactome hyaluronan biosynthesis and export, and nfkb pathway (Fig. 1L-M). These results suggested that CRISPR/Cas9 screening technology indeed provides a large number of reliable differential genes for promoting or inhibiting CRC liver metastasis, which can be targeted for subsequent studies.

### CHSY1 was overexpressed in CRC primary and liver metastasis tissues that indicated a worse clinical prognosis

Based on the results of CRISPR/Cas9 technology, we screened some differential genes in the negative group for targets that promote CRC liver metastasis, and initially found that the CHSY1 gene may be closely associated with liver metastasis of CRC (Fig. 1E). However, this result was based on a mouse model. To verify that CHSY1 is indeed also expressed in liver metastatic tissues of human CRC, single-cell RNA sequencing was used to detect primary CRC, adjacent normal intestinal tissues, CRC liver metastases, adjacent normal liver tissues, and preoperative blood samples. Using the UMAP map, specific genetic markers are defined by taxonomy (Figure S2A-B), from which we obtained 12 cell clusters including: cancer cells, natural killer (NK) cells, monocytes, B lymphocytes (B cells), epithelial cells cells, cancer associated fibroblasts (CAF), endothelial cells, plasma, myofibroblasts, progenitor cells, T cells and tumor associated macrophages (TAM). For example, endothelial cell clusters express VWF, CDH5, etc., while CAF clusters specifically express COL1A1, DCN, etc.(Figure S2B). Bar and violin plots showed relatively high expression of CHSY1 in the total sample analysis of cancer cell clusters (Figure S2C-D). According to dot plots, CHSY1 was significantly upregulated in the cancer cell population of primary CRC tissue compared to samples from different parts of the body. In addition, it was highly expressed in metastatic foci of CRC compared to paracancerous tissue (Figure S2E).

TCGA results showed that CHSY1 was significantly overexpressed in CRC tissues compared with the corresponding paraneoplastic tissues (Fig. 1G). Next, CHSY1 expression was detected by qRT-PCR in 54 human CRC tissues and adjacent normal tissues, and CHSY1 was found to be significantly overexpressed in CRC tissues compared with the corresponding paraneoplastic tissues (Fig. 1H). According to the clinicopathological characteristics, CHSY1 overexpression was positively correlated with TNM classification and lymph node metastasis, while there was no significant correlation with age, gender, tumor size and differentiation (Table 1). According to Kaplan–Meier survival curves, overall survival was reduced in patients with CRC and high CHSY1 expression (Fig. 1I). This information confirms that CHSY1 is indeed highly enriched in human CRC liver metastasis samples, which is consistent with the results of CRISPR/Cas9 screening in mice.

**Table 1:** Clinical characteristics of 54 patients with CRC

Characteristic	Total (cases [%])	CHSY1 expression (cases [%])		*P* value
		**Low (*n* = 32)**	**High (*n* = 22)**	
**Age**				0.295
< 65 years	21	11	10	
≥ 65 years	33	21	12	
**Gender**				0.205
Male	32	17	15	
Female	22	15	7	
**Tumor size**				0.520
< 5 cm	28	17	11	
≥ 5 cm	26	15	11	
**Differentiation**				0.684
High-middle	10	6	4	
Low	44	26	18	
**TNM stage**				< 0.001
I-II	33	29	4	
III-IV	21	3	18	
**Lymph node metastasis**				< 0.001
Absent	32	29	4	
Present	22	3	18	

### CHSY1 facilitated the liver metastasis of CRC in vitro and in vivo

**Fig. 2:**
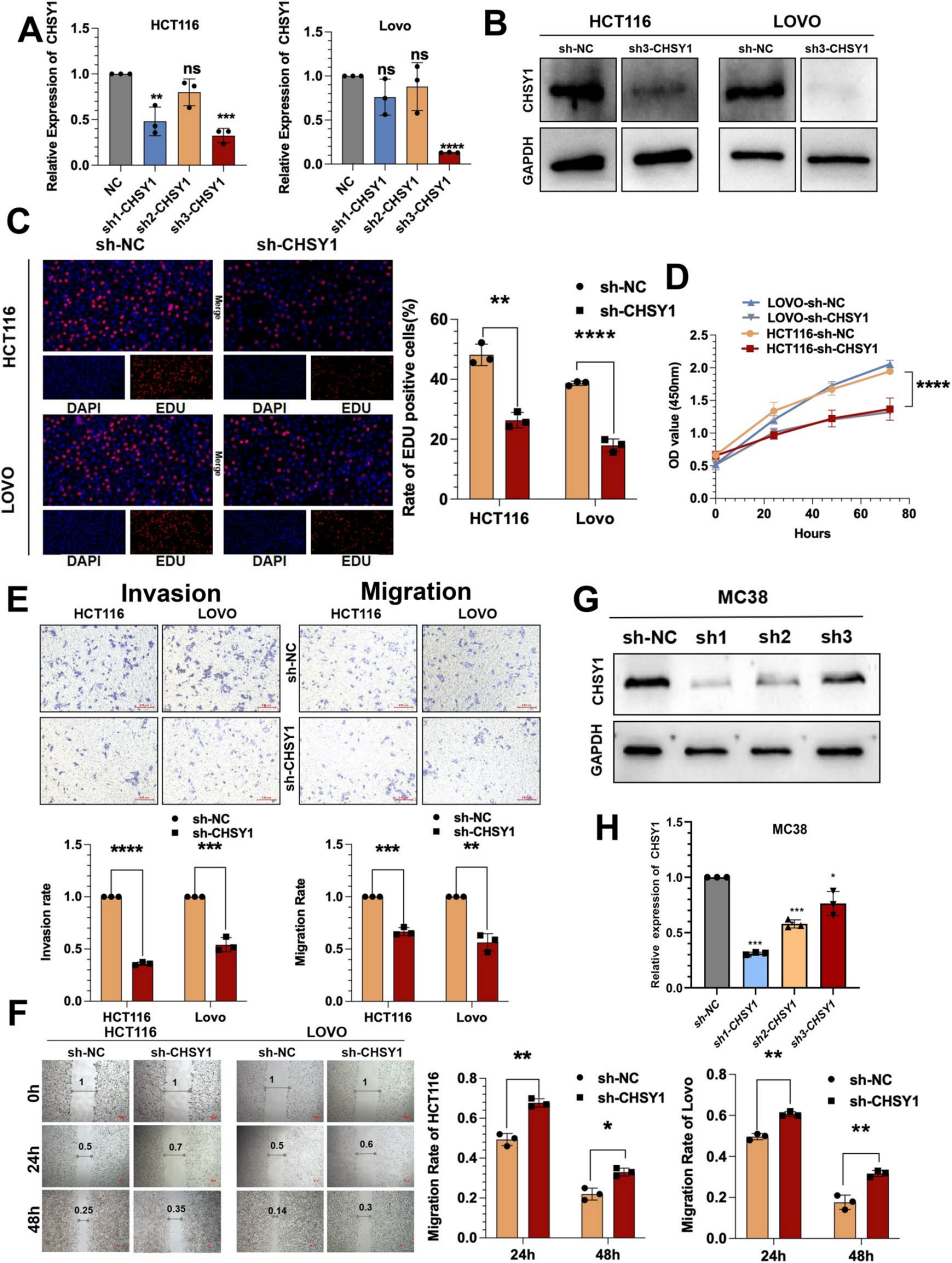
CHSY1 facilitates the proliferation, invasion and migration of CRC cells. **A**, **B** Three shRNAs (sh1, sh2, and sh3) were designed to silence CHSY1 in CRC cells (HCT116 and LOVO), and validated by qRT-PCR and western blotting. **C** EdU assay was performed to assess cell proliferation of CRC cells transfected with sh3-CHSY1/sh-NC. **D** The growth curves of cells were plotted after transfection with sh3-CHSY1/sh-NC based on CCK-8 assay. **E** Transwell invasion and migration assay were performed to assess invasion and migration of CRC cells transfected with sh3-CHSY1/sh-NC.Scale bar, 100 μm. **F** Wound Healing assay was performed to assess migration of CRC cells transfected with sh3-CHSY1/sh-NC.Scale bar, 100 μm. **G**-**H**Three shRNAs (sh1, sh2, and sh3) were designed to silence CHSY1 in MC38 cells, and validated by qRT-PCR and western blotting. *, *P* < 0.05; **,* P* < 0.01; ***,* P* < 0.001;****,* P* < 0.0001

However, the role of CHSY1 in human CRC cells deserves further exploration. Three shRNAs targeting CHSY1 were developed to silence CHSY1 in HCT116 and LOVO cells and assessed the gene repression efficiency using qRT-PCR. We found that only sh3-CHSY1 (sh-CHSY1) could synchronously knock down two cell lines, so we used it as a subsequent knock down sequence and validated its expression at the protein level via western blotting(Fig. 2A-B). sh-CHSY1 inhibited proliferation of HCT116 and LOVO cells, as per the results of CCK-8 and EdU assays (Fig. 2C-D). Transwell and wound healing assays showed that sh-CHSY1 significantly hindered the invasion and migration functions of HCT116 and LOVO cells (Fig. 2E-F). In contrast, the CHSY1 overexpression model exerted the opposite effect (Figure S3A-F).

To explore the relevance of CHSY1 to CRC growth and metastasis in vivo, shRNA against CHSY1 in MC38 cells was confirmed by qRT-PCR and western blotting (Fig. 2G-H). The sh1-CHSY1(sh-CHSY1) and sh-NC MC38 cells were injected into the spleen of C57BL/6 mice, respectively (Fig. 5A). The down-regulated expression of CHSY1 in the sh-CHSY1 group significantly inhibited the liver metastasis of CRC compared to the sh-NC group (Fig. 5B-C). According to the immunohistochemical results, Ki-67 expression in liver metastasis tissues was significantly decreased in the sh-CHSY1 + PBS group (Fig. 5D-E). In conclusion, these results suggest that inhibition of CHSY1 can reduce liver metastasis of CRC in vitro and in vivo.

### CHSY1 induced CD8^+^ T cell exhaustion and upregulated PD-L1 expression in vitro and in vivo

**Fig. 3:**
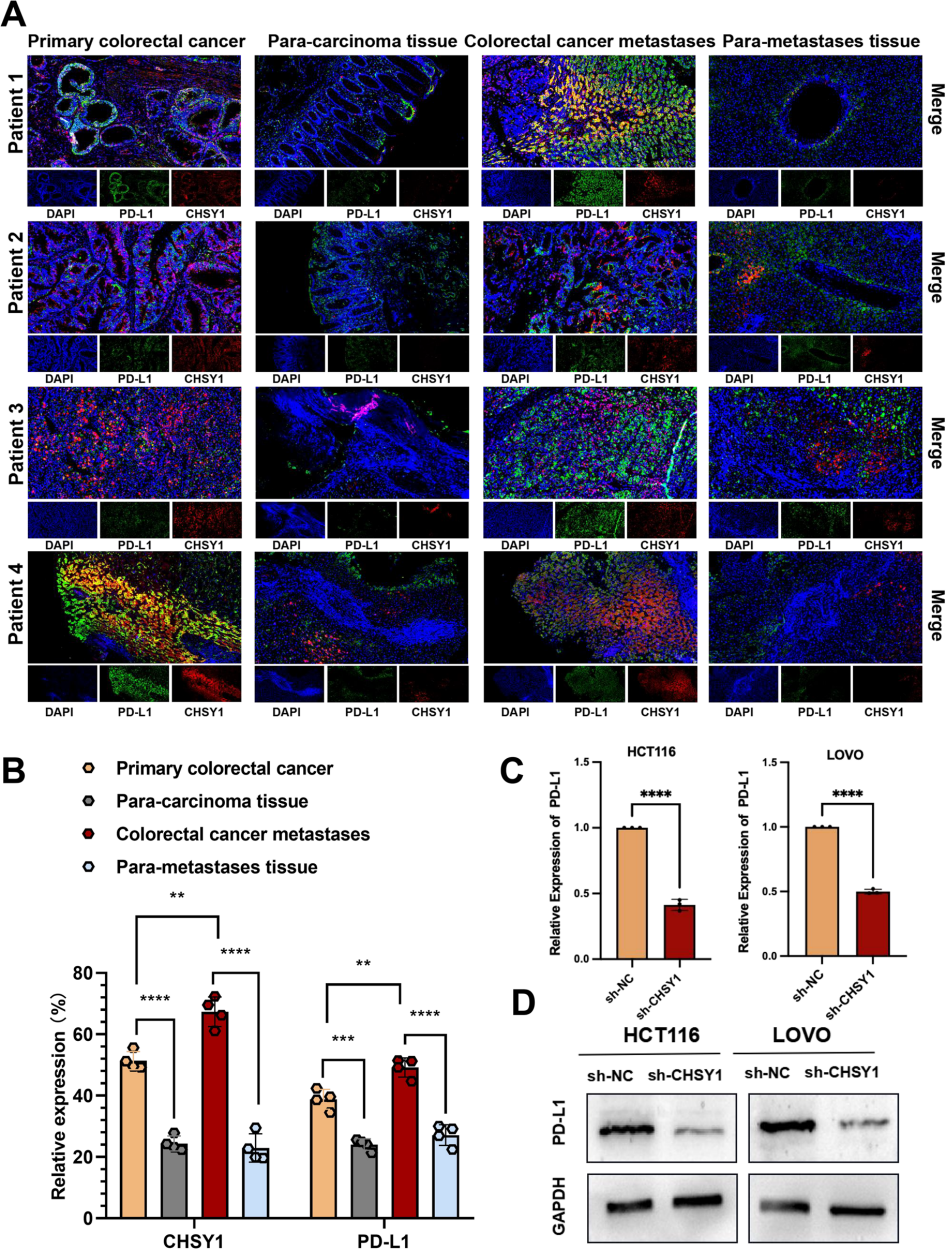
Expression profiles of CHSY1 and PD-L1 in human tissue samples and their interactions in human CRC cell lines. **A**, **B** Immunofluorescence detection of CHSY1 and PD-L1 expression in primary and secondary lesions and corresponding paracancerous tissues of 4 patients with CRC liver metastases. **C**, **D** qRT-PCR and western blotting were performed to detect PD-L1 expression in human CRC cells after CHSY1 knockdown.**,* P* < 0.01; ***,* P* < 0.001;****,* P* < 0.0001

The above results raised our curiosity about the reason why CHSY1 promotes CRC liver metastasis. We further analyze CRISPR/Cas9 screening sequencing data from mouse colorectal cancer liver metastasis models and compare differential genes (positive and negative) between control and experimental groups and surprisingly found differential genes associated with interleukin 2, interleukin 15, TGFb1, neutrophils, HIF1ɑ, inflammatory response, TAL1, and B lymphocytes (Fig. 1L-M), suggesting that liver metastasis of CRC might be closely related to tumor immunity. We used TISIDB database to predict and analyze the correlation between CHSY1 expression and immune factors including PD-L1, PD1, LAG3, IDO1 and CTLA4, so that we could observe the interaction between CHSY1 and the CRC tumor microenvironment. The results showed that there was a significant positive correlation between CHSY1 expression and the expression of immunosuppressive markers such as PD-L1, PD1, LAG3, IDO1 and CTLA4) (Figure S4). Based on the fact that PD-L1/PD1 is the most popular target and has been routinely used in clinical practice, we selected PD-L1 as a downstream gene of CHSY1 in cancer cells. The primary and secondary lesions and corresponding paracancerous tissues of four patients with CRC liver metastases were first detected by immunofluorescence, and the results showed that CHSY1 expression in cancer tissues was higher than that in paracancerous tissues. The expression of CHSY1 was higher in the metastatic foci than in the primary lesions. The same trend of PD-L1 expression was observed, which seemed to indicate a relationship between CHSY1 and PD-L1 expression (Fig. 3A-B). In order to observe the changes of PD-L1 protein and mRNA expression in cells after CHSY1 knockdown, we performed qRT-PCR and western blotting experiments on HCT116 cells and LOVO cells of different groups (sh-NC group and sh-CHSY1 group). The results showed that PD-L1 protein and mRNA expression decreased with the decrease of CHSY1 expression (Fig. 3C-D).

**Fig. 4:**
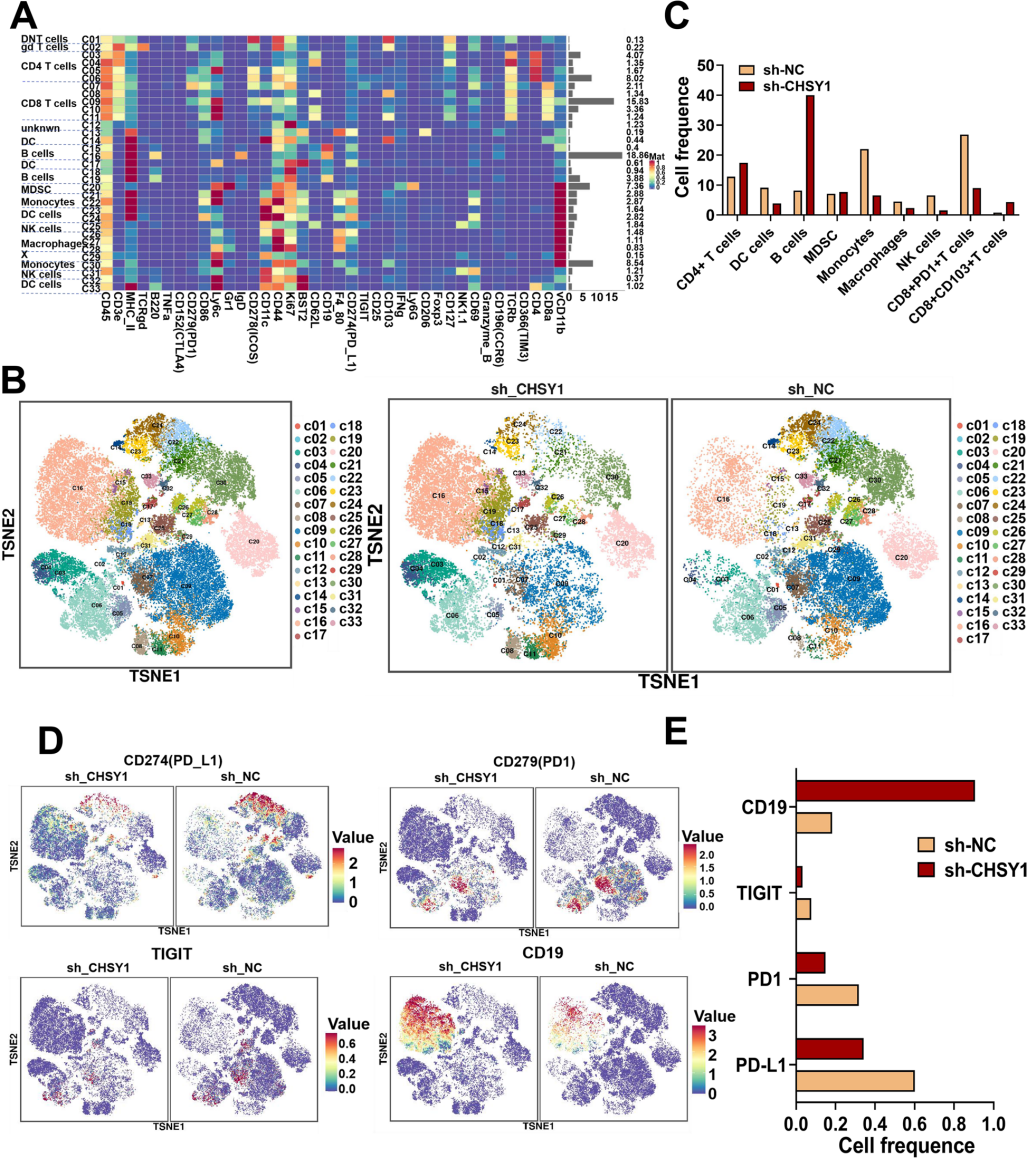
CHSY1 induced CD8^+^ T cell exhaustion and upregulated PD-L1 expression. **A** A total of 33 cell clusters were divided, and we defined the respective group of the mass cytometry results. **B** TSNE plot showing distribution of 33 cell clusters in the respective sample. **C** The histogram showing the number of the respective cell clusters in different groups by mass cytometry. **D** TSNE plot showing distribution of PD1, PD-L1, TIGIT, and CD19 in two groups. **E** The histogram showing the expression of PD1, PD-L1, TIGIT, and CD19 in different groups

**Fig. 5:**
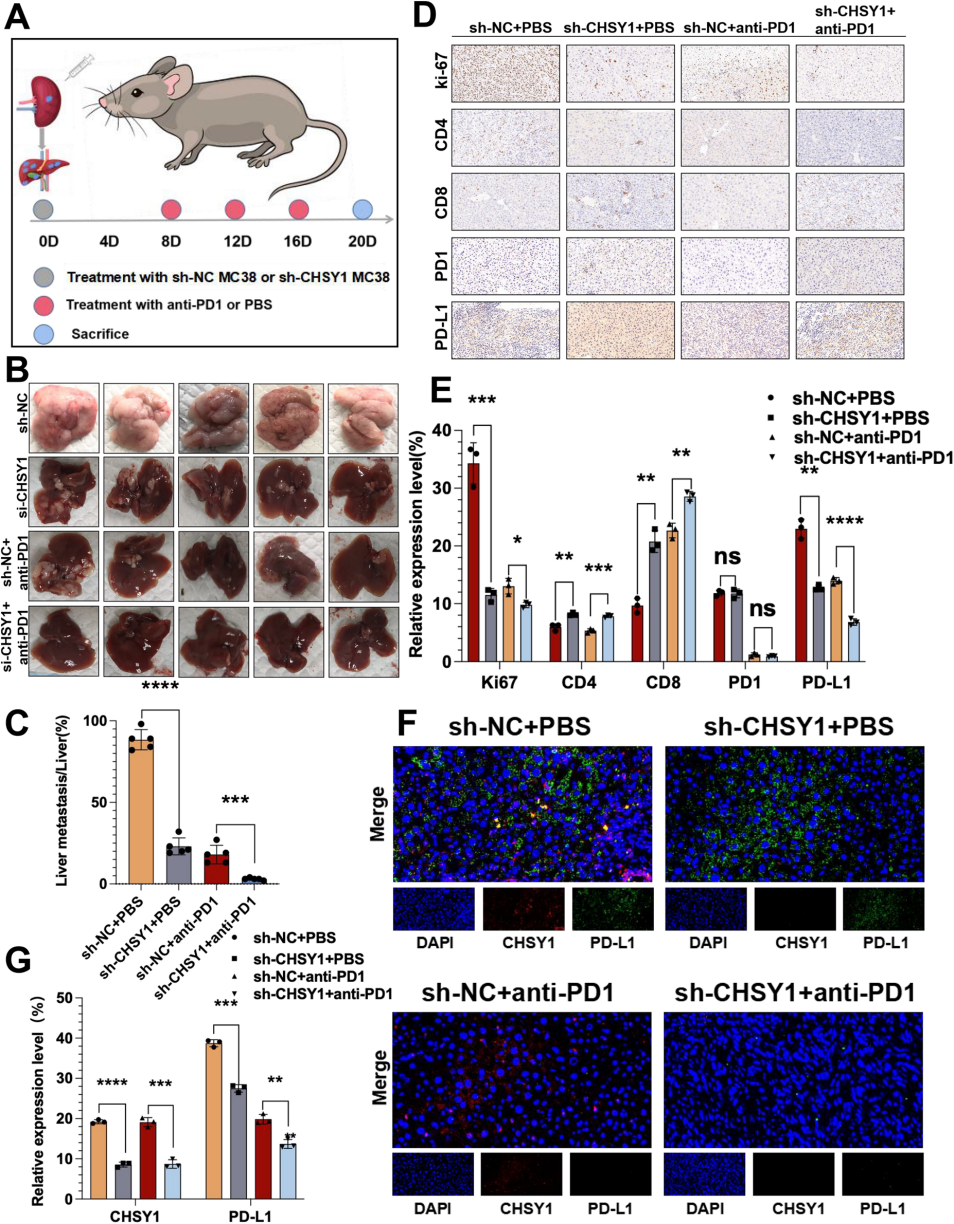
The anti-tumor effect of knockdown CHSY1 combined with anti-PD1 in vivo. **A** Schematic diagram of the establishment of a mouse model of CRC liver metastases. **B** Images of liver metastases in each group. **C** Analysis of liver metastases tumors in the respective groups. (multiple unpaired t test) (**D**, **E**) Immunohistochemistry results of CD8, CD4, Ki67, PD-L1 and PD1 expression in the respective groups. **F**, **G** The immunofluorescence was used to detect the expression of CHSY1 and PD-L1 in liver metastases in each group. *, *P* < 0.05; **,* P* < 0.01; ***,* P* < 0.001;****,* P* < 0.0001.ns indicates no statistical difference

Since human samples and cellular experiments cannot adequately simulate the tumor microenvironment, we demonstrated in vivo that CHSY1 promoted CD8^+^ T cell exhaustion and liver metastasis from CRC. We further evaluated the effect of CHSY1 expression on the immune microenvironment of liver metastases by mass spectrometry. Single, live, intact CD45^+^ immune cells were extracted from CRC liver metastases in the sh-CHSY1 + PBS group and the sh-NC + PBS group. The cell clusters were determined according to specific markers, and finally 33 cell clusters were obtained (Fig. 4A-C, Figure S5). According to the results, B cells and CD4^+^ T cells were increased, while NK cells, monocytes, and macrophages decreased after downregulation of CHSY1 (Fig. 4B-C). We further identified CD8^+^ T cell subsets in detail and were surprised to find a decrease in CD8^+^ PD1^+^T cells and an increase in CD8^+^103^+^T cells (Fig. 4B-C). When CHSY1 was downregulated, the expression of common markers of CD8^+^ T cell exhaustion (for example, PD-L1, PD1, TIGIT) was decreased and CD19 was upregulated (Fig. 4D-E). Immunohistochemical results from in vivo experiments further validated that knockdown of CHSY1 increased the expression of CD8^+^ T cells and decreased the expression of PD-L1 in the tumor microenvironment (Fig. 5D-E). Thus, knockdown of CHSY1 suppressed CD8^+^ T cell exhaustion and enhanced the expression of CD4^+^ T and CD8^+^103^+^T cells to kill cancer. These results suggest that the combination of CHSY1 knockdown and anti-PD1 may further enhance the effect of inhibiting CRC liver metastasis.

To confirm the anti-tumor effect of knockdown CHSY1 combined with anti-PD1 in vivo, MC38 cells were injected into the spleen of C57BL/6 mice in the sh-CHSY1 and sh-NC groups, respectively, followed by anti-PD1 injection to assess the liver metastatic ability of these cells (Fig. 5A). When anti-PD1 was also used, the liver metastasis inhibition rate was significantly higher in the sh-CHSY1 + anti-PD1 group compared with the sh-NC + anti-PD1 group (Fig. 5B-C). Immunohistochemical results showed a significant decrease in Ki-67 and PD-L1 expression and a significant increase in CD8 infiltration in the sh-CHSY1 group, and this trend was more pronounced after the combination of anti-PD1 (Fig. 5D-E). The results of immunofluorescence verified the expression of CHSY1 and PD-L1 (Fig. 5F-G). Taken together, these results show that inhibition of CHSY1 can reduce liver metastasis and improve the efficiency of anti-PD1 treatment for CRC.

### Metabonomics analysis of down-regulating CHSY1 in CRC cells

**Fig. 6:**
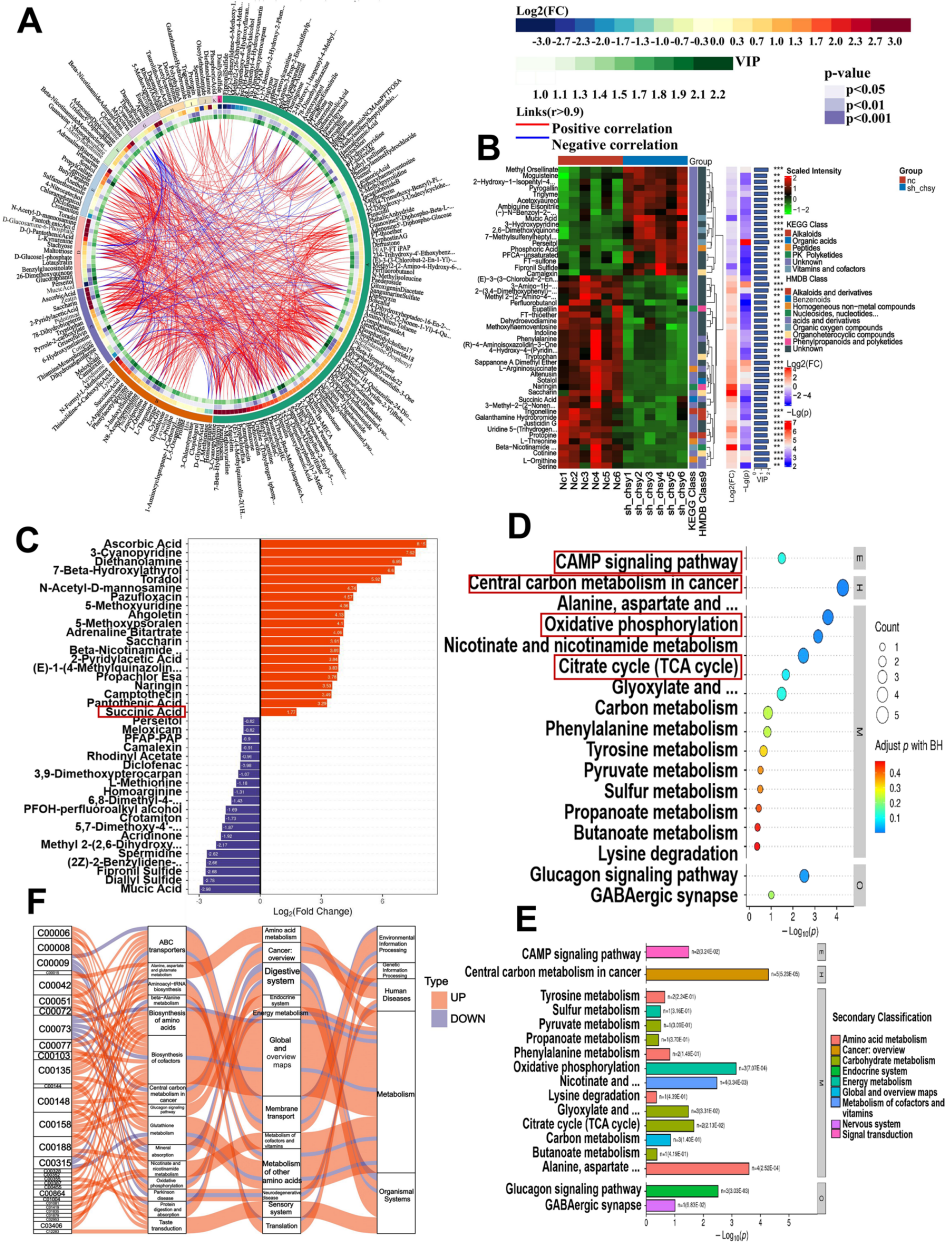
Metabonomics analysis of down-regulating CHSY1 in CRC cells. **A** circos plot showing correlations between multiple differential metabolites. **B** According to the structure and function of the metabolites, the different metabolites in each control group were classified and counted, and the classification results of the substances in the KEGG and HMDB databases were provided respectively. **C** Top 20 up-regulated metabolites and top 20 down-regulated metabolites. **D**, **E** KEGG pathway analysis indicated the main concentrated pathways of these differential metabolites. **F** Sankey diagram visualization of data flow trends between down-regulated metabolites and various pathways

The above in vitro and in vivo results confirmed that CHSY1 promotes CD8 ^+^ T cell exhaustion and causes upregulation of PD-L1 in particular, thus its mechanism deserves further exploration. Metabolomics techniques allow for precise analysis of metabolites as well as comprehensive and accurate studies of cytokines and signaling pathways. We infected CRC cell line LOVO with lentvirus targeting CHSY1 expression. After determining that CHSY1 expression was knocked down in LOVO cells, we performed metabolomic sequencing using sh-NC LOVO cells and sh-CHSY1 LOVO cells, each containing six samples. In this study, CRC cells from the sh-NC and sh-CHSY1 groups were detected by metabolomics sequencing. We used negative, positive and mixed modes to analyze different metabolites, respectively. The mixed mode was applied first. The cycle diagram mainly showed the correlation between multiple differential metabolites (Fig. 6A). The different metabolites in each comparison group were classified and counted according to the structure and function of the metabolites, and the results of substance classification in the KEGG and HMDB databases were provided respectively (Fig. 6B). The top 20 up-regulated metabolites (including perseitol, meloxicam, PFAP-PAP, camalexin, rhodinyl acetate, diclofenac, etc. The top 20 down-regulated metabolites (including propachlorEsa, naringin, camptothecin, pantothenic acid, and succinic acid, etc. were shown by multiplicity of differences in Fig. 6C. KEGG pathway analysis showed that these differential metabolites were mainly focused on the CAMP signaling pathway, Central carbon metabolism in cancer, Oxidative phosphorylation and Citrate cycle (TCA cycle), etc. (Fig. 6D-E). A visual analysis of data flow trends between different metabolites and various pathways using Sankey plots showed that the largest pool of differential metabolites in the rightmost hierarchy was in metabolism, while the largest concentration in metabolic pathways was in the global and overview plots of amino acid and cofactor biosynthesis (Fig. 6F). Next, both positive and negative patterns were analyzed and the results are shown in Figure S6-S7. The results of the metabolomic analysis indicated that the metabolites are significantly altered after CHSY1 knockdown.

### CHSY1 promoted CD8^+^ T cell exhaustion by activating the succinate metabolism pathway leading to liver metastasis of CRC

**Fig. 7:**
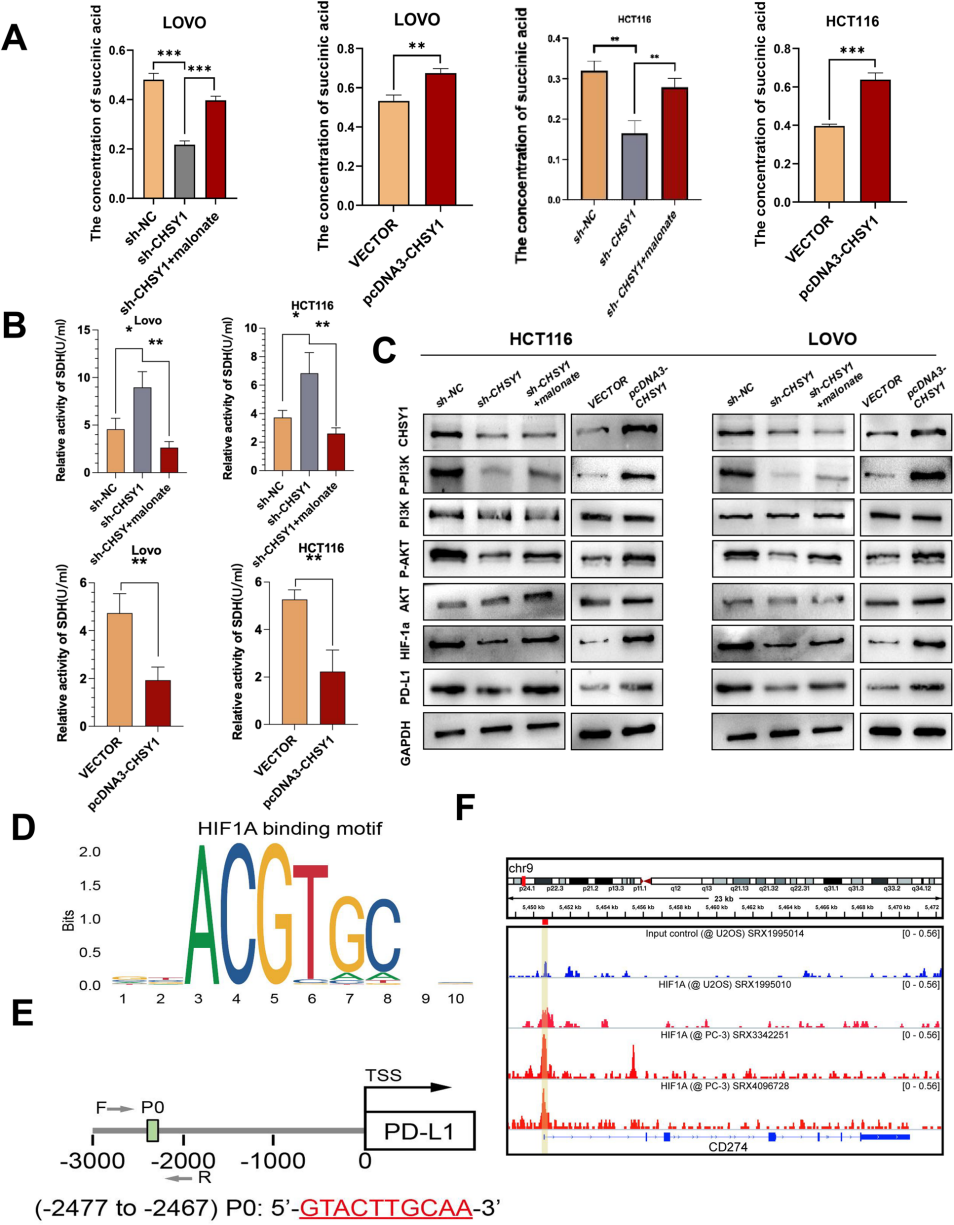
CHSY1 affect PD—L1 expression and cause of CRC liver metastasis through SDH/succinate/PI3K/AKT/HIF1A pathways. **A** Determination results of succinic acid content in each group of samples. **B** Determination results of SDH concentration in each group of samples. **C** Western blotting results of the expression of P-PI3K, P-AKT, HIF1A and PD-L1 in different groups. **D**, **E** The JASPAR database predicted that the PD-L1 gene promoter covered the HIF1A binding site. **F** ChIP data of GSE85096 and GSE85096 showed that HIF1A has a prominent peak upstream of PD-L1. *, *P* < 0.05; **,* P* < 0.01; ***,* P* < 0.001

Analysis of three models showed that knockdown of CHSY1 resulted in a significant reduction in succinate and pathway enrichment including the cAMP signaling pathway [11], oxidative phosphorylation [12] and the citric acid cycle (TCA cycle) [13]. These are all closely related to succinate metabolism. Therefore, succinate was selected as a downstream target of CHSY1. We first examined the changes in succinate content in the sh-NC, sh-CHSY1, ov-NC and ov-CHSY1 groups by mass spectrometry and found that succinate content decreased after CHSY1 knockdown, whereas the opposite result was observed after CHSY1 increase (Fig. 7A). Previous studies have shown that succinate dehydrogenase (SDH) catalyzes the conversion of succinate to fumarate in the Krebs cycle and is thought to be critical in controlling succinate levels in mitochondria. Defects in SDH in cancer cells lead to abnormal accumulation of succinate in the mitochondria and cytoplasm [14]. Therefore, we wanted to know whether SDH activity would be affected by CHSY1 knockdown. Malonic acid, an inhibitor of SDH activity, inhibits intracellular SDH activity at a concentration of 2 nM. The results of the SDH activity assay showed that knockdown of CHSY1 significantly increased SDH activity in LOVO and HCT116 cell lines, while the opposite result was observed for overexpression of CHSY1. Furthermore, the addition of malonate after knockdown of CHSY1 could inhibit SDH activity again (Fig. 7B). Also, we tested the addition of malonate to sh-CHSY1 cells and showed that the addition of malonic acid restored the decrease in succinate caused by knockdown of CHSY1 (Fig. 7A). These results suggest that CHSY1 regulates succinate metabolism by affecting the activity of SDH.

Then the effect of CHSY1 on downstream genes after activation of the succinate pathway deserves further investigation. We enriched the CRISPR/Cas9 results and surprisingly found that differential genes were associated with PI3K/AKT pathway, HIF1α targets, TGFβ1 targets, Hypoxia, etc. (Fig. 1L). Excitingly, previous studies reported that succinate could stabilize HIF-1a expression and promote lung cancer cell migration through the PI3K/AKT signaling pathway [15]. Furthermore, we explored whether CHSY1 could activate the PI3K/AKT/HIF1A pathway and thus affect the expression of PD-L1. Interestingly, we found that downregulation of CHSY1 expression could inhibit the expression of P-PI3K, P-AKT, HIF1A and PD-L1, while overexpression of CHSY1 showed the opposite results (Fig. 7C). According to the JASPAR database, the PD-L1 gene promoter was predicted to cover the HIF1A binding sites (Fig. 7D-E). ChIP data for GSE85096 and GSE85096 showed a significant peak of HIF1A upstream of PD-L1, suggesting a clear binding site between HIF1A and PD-L1 (Fig. 7F). The above results suggest that CHSY1 leads to CD8^+^ T cell exhaustion through activation of succinate metabolism and PI3K/AKT/HIF1A pathway in CRC liver metastasis.

### Artemisinin as a CHSY1 inhibitor reduced liver metastasis and enhance the effect of anti-PD1 in CRC

**Fig. 8:**
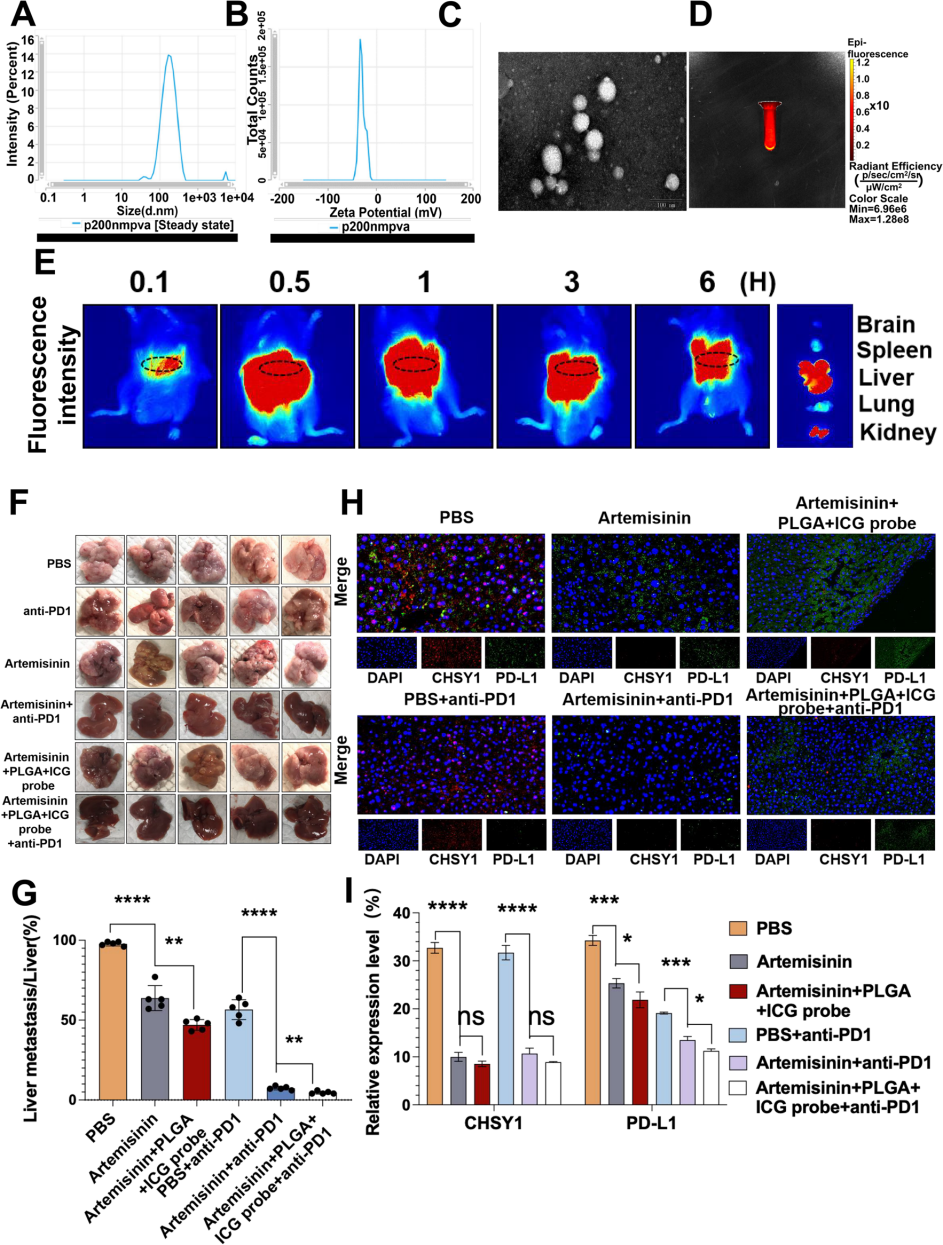
PLGA-loaded Artemisinin and ICG probe as a CHSY1 inhibitor can reduce liver metastasis and enhance the effect of anti-PD1 in CRC liver metastasis. **A** DLS potential of Artemisinin-coupled fluorescence on PLGA. **B** Zeta potential of Artemisinin-coupled fluorescence on PLGA. **C** PLGA-loaded Artemisinin coupled fluorescence display. **D** Fluorescence imaging of PLGA loaded Artemisinin with ICG probe. **E** Metabolic distribution of PLGA-loaded Artemisinin-linked ICG probe in mice. **F** Images of liver metastases in each group. **G** Analysis of liver metastases tumors in the respective groups. (**H**-**I**) The immunofluorescence was used to detect the expression of CHSY1 and PD-L1 in liver metastases in each group.*, *P* < 0.05; **,* P* < 0.01; ***,* P* < 0.001;****,* P* < 0.0001.ns indicates no statistical difference

As there is no readily available drug to inhibit CHSY1, we searched the literature and unexpectedly found that Artemisinin could inhibit CHSY1 expression [16]. Molecular docking was used to verify the binding activity between the active ingredient and the key target. The binding energy between artemisinin and CHSY1 is -8.0 kcal/mol. In general, if the binding energy between the ligand and the target protein is less than 0, the ligand and the receptor protein can spontaneously bind, if the binding energy is <  − 5.0 kcal/mol, it indicates that the active ingredient has a strong affinity with the target, so the binding between the small molecule artemisinin and the CHSY1 target protein is stable. According to the results of the three-dimensional diagram(Figure S9A-C), artemisinin can bind to the 429 PHE amino acid residue, 410 MET amino acid residue, 295 SER amino acid residue and 409 VAL amino acid residue of the receptor protein CHSY1 through hydrophobic forces. The results showed that artemisinin could bind to CHSY1 stably. qPT-PCR and western blotting confirmed that Artemisinin could inhibit CHSY1 expression in CRC cells (Figure S8A-B). According to the results of CCK-8, EdU, Transwell and wound healing assays, artemisinin inhibited the proliferation, invasion and migration of HCT116 and LOVO cells (Figure S8C-F). In vivo experiments confirmed that Artemisinin inhibited liver metastasis of CRC compared with the PBS group, and the rate of liver metastasis inhibition was significantly higher in the Artemisinin + anti-PD1 group than in the PBS + anti-PD1 group (Fig. 8F-G). Immunohistochemical results showed that artemisinin significantly decreased the expression of Ki-67 and PD-L1 and significantly increased the infiltration of CD8 T cells, and the trend was more obvious with the combination of anti-PD1 (Figure S9D-E). Immunofluorescence results showed that Artemisinin decreased the expression of CHSY1 and PD-L1 in tumor tissues, while the combination of anti-PD1 increased this trend (Fig. 8H-I).

### PLGA-loaded Artemisinin and ICG probe reduced liver metastasis and increased the efficiency of anti-PD1 treatment in CRC

In order to further improve the utilization of Artemisinin and visualize the drug metabolism, we constructed a PLGA as a carrier to encapsulate Artemisinin and fluorescence in PLGA. The DLS and Zeta potentials of Artemisinin-coupled fluorescence on PLGA were detected, and the Zeta potential was -29.02 mV. Average particle size: 181.6 nm; Intensity: 188.4 nm; Number: 89.98 nm; PDI: 0.3109 (Fig. 8A-B). Figure 8C and D show the fluorescence imaging of PLGA-loaded Artemisinin couples and PLGA-loaded Artemisinin couples with ICG probes, respectively. After injection of PLGA-loaded Artemisinin coupling into the tail vein of mice, we observed that the drug concentration peaked in the liver after 0.5 h, while the intravenously injected drug rarely aggregated and metabolized in the liver and kidneys (Fig. 8E).

To investigate the relevance of PLGA-loaded Artemisinin and ICG probe to the antimetastatic effect of CRC in vivo, the spleen of C57BL/6 mice was injected with MC38 followed by anti-PD1 in the PLGA-loaded Artemisinin + ICG probe group and the Artemisinin group, respectively, to assess these cells' hepatic metastatic capacity. The PLGA-loaded Artemisinin + ICG probe significantly inhibited liver metastasis of CRC, compared with Artemisinin group. In addition, when anti-PD1 was also administered, the PLGA-loaded artemisinin + ICG probe group showed significantly higher inhibition of liver metastasis compared to the Artemisinin + anti-PD1 group (Fig. 8F-G). Immunohistochemical results showed that PLGA-loaded Artemisinin + ICG probe significantly decreased Ki-67 and PD-L1 expression than Artemisinin alone, and also significantly increased CD8^+^ T cell infiltration, with a more pronounced trend of combined anti-PD1 (Figure S9D-E). Immunofluorescence results showed that the PLGA-loaded Artemisinin + ICG probe decreased CHSY1 and PD-L1 expression in tumor tissues more significantly than Artemisinin alone (Fig. 8H-I). Overall, PLGA-loaded Artemisinin and ICG probes reduced liver metastases and significantly improved the efficacy of anti-PD1 treatment for CRC.

## Discussion

Since its first use as a genome editing tool in mammalian cells in 2013 [17, 18], the toolbox of CRISPR/Cas9 has been expanded to not only modify the genome sequence of cells and organisms, but also to introduce epigenetic and transcriptional modifications. CRISPR/Cas9 and improved versions have been widely used for genome engineering and activation or repression of gene expression. Thus, large-scale CRISPR/Cas9 screens have been performed to systematically identify potential genes in many cancer cell lines [19–21]. Previous studies have used genome-wide CRISPR/Cas9 to screen pancreatic ductal adenocarcinoma cells with RNF43 mutations. The results suggest that FZD5 antibody is a potential targeted therapy [22]. Through a genome-wide CRISPR screen, Michael A et al. [23] found that ENL is necessary for the growth and proliferation of MLL-AF4-positive acute myeloid leukemia(AML) cell lines. However, our use of CRISPR/Cas9 to screen for liver metastasis in CRC is the first report in the world, and we screened for the CHSY1 gene as a possible culprit in promoting liver metastasis.

Recent studies have reported that CHSY1 was overexpressed in various tumors (for example, hepatocellular carcinoma [24], glioma [25], soft tissue sarcomas[26]), demonstrating its possible association with tumor progression. Liu et al.[24] reported that CHSY1 could be involved in the induction of malignant behavior of cells through the regulation of hedgehog malignant behavior of hepatocellular carcinoma cells. Wu et al.[27] reported that CHSY1 was associated with poorer prognosis in human CRC, but no one has studied the function and mechanism of CHSY1 in cancer in depth. Our study validated by single-cell RNA sequencing analysis and qRT-PCR, showed that CHSY1 was overexpressed in CRC primary and liver metastatic tissues, indicating a poor clinical prognosis. Inhibition of CHSY1 reduced liver metastasis of CRC in vitro and in vivo. Our study provides a new target for liver metastasis of CRC.

Immune checkpoint inhibitors (ICI) including the use of anti-PD1, have become the standard therapy for the treatment of metastatic CRC. CRC is classified into mismatch repair-deficient and microsatellite instability-high (dMMR-MSI-H) features and mismatch repair-effective and microsatellite instability-low features [28–30]. However, dMMR-MSI-H CRC responded poorly to ICI immunotherapy [31, 32]. One of the reasons for uncontrolled tumor growth is the induction of CD8^+^ tumor-infiltrating T cells exhaustion [33–36]. Chen and Mellman [37] classified the tumor immune microenvironment into three basic types. Immune desert phenotypes, immune -rejection tumors, and inflamed tumors. Based on this classification, the effectiveness of PD-L1 inhibitors depends on the expression of PD-L1 in the tumor and the presence of sufficient immune effector cells in the tumor microenvironment (TME), particularly CD8^+^ T cells. De Smedt et al. [38] found that in MSI (Microsatellite instability)-H CRC patients benefiting from pembrolizumab immunotherapy, tumor infiltrating CD4^+^ and CD8^+^ T cells were significantly higher than those of MSS (microsatellite stable) CRC. How to reverse the process of T-cell depletion is a major challenge for immunotherapy. In the present study, we enriched the results of CRISPR/Cas9 and surprisingly found that different genes were associated with T cell responses. To assess the changes in the immune microenvironment of liver metastatic tissues in the sh-CHSY1 + PBS and sh-NC + PBS groups, we measured the expression of the respective immune cell populations by mass spectrometry. According to the results, we were surprised to find that CD8^+^ CD103^+^ T cells and B cells were increased after downregulation of CHSY1. CD8 tissue resident memory T (TRM) cells marked by CD103 (ITGAE) expression are believed to actively inhibit cancer progression, and intra-tumor CD8^+^ CD103^+^ T cells can predict the response to PD-L1 blockade [39], including in bladder cancer [40] and lung cancer [41], and are associated with good prognosis of patients. When CHSY1 was downregulated, the expression of common markers of CD8^+^ T cell failure was significantly reduced. Thus, knockdown of CHSY1 inhibited CD8^+^ T cell failure and enhanced the expression of CD103^+^CD8^+^ T cells and B cells to kill CRC. However, this still needs to be further verified by subsequent large samples.We also demonstrated that inhibition of CHSY1 reduced liver metastasis and improved the efficiency of anti-PD1 treatment for CRC. Our study provides important evidence on how to improve the effectiveness of anti-PD1 in CRC.

The above mass spectrometry results comprehensively reflected the effect of CHSY1 knockdown on the tumor immune microenvironment. In this study, we also elaborated the molecular mechanisms by which CHSY1 promotes PD-L1 expression in CRC cells and leads to CD8^+^ T cell efflux. Metabolomics technologies enable precise analysis of metabolites and comprehensive and accurate studies of cytokines and signaling pathways. In this study, CRC cells in the sh-NC and sh-CHSY1 groups were examined by metabolomic sequencing. Analysis of metabolomics showed that knockdown of CHSY1 resulted in a significant reduction of succinate and that enrichment of pathways were all closely associated with succinate metabolism. We then demonstrated at the cellular level that CHSY1 leads to CD8^+^ T cell depletion through activation of succinate metabolism and PI3K/AKT/HIF1A pathway in CRC liver metastases. Recently, it has been reported that succinate metabolism plays an important role in various tumors.Tao et al. [42] reported that a decrease in SDHA/B(Succinate dehydrogenase subunit A/B) promotes HCC proliferation.SDHB silencing leads to increased levels of HIF-1α and adenosine monophosphate-activated protein kinase, which promotes ovarian cancer metastasis [43]. Studies have shown that SDHB and SDHD(Succinate dehydrogenase subunit D) [44] mutations are present in renal cell carcinoma and thyroid tumors. Increased succinate and expression of HIF1α and angiogenic genes, such as VEGF, as well as high density of microvessels were found in pheochromocytoma tumor tissues [45]. After these observations confirmed its role in the development of certain tumors, SDH was defined as a tumor suppressor and succinate as a parametabolite [46]. To our knowledge, this is the first study reporting CRC liver metastasis, i.e., CHSY1 promotes CRC liver metastasis through activation of the succinate metabolic pathway.The role of CHSY1 in CRC has been reported, but not in relation to liver metastasis. For instance, Zeng et al. [47] found that CHSY1 regulates tumor growth by modulating NF-κB signaling and Caspase-3/7 signaling in CRC. Our previous study indicated that Matrix Gla protein(MGP) could facilitate CD8^+^ T cell depletion through activation of the NF-κB pathway, which leads to liver metastasis of CRC [48]. The present study innovatively demonstrated that CHSY1 can promote CD8^+^ T cell depletion and lead to CRC liver metastasis through activation of the succinate metabolic pathway, providing new clues for immunotherapy of CRC liver metastasis.

In order to achieve molecular targeting and clinical translation of CHSY1, we innovatively discovered that Artemisinin could inhibit CHSY1 activity. Artemisinin is an active compound that has been used to treat fever for more than 2000 years [49]. Artemisinin and its derivatives are important first-line drugs against simple malaria infections [50, 51]. They are effective in eliminating multidrug-resistant Plasmodium falciparum because of their potent efficacy and few side effects [52]. In in vitro and in vivo animal studies, the active compounds of artemisinin and its derivatives have been shown to have cytotoxic effects on a variety of human cancer cells [53–57]. Wang et al. [58] demonstrated the effectiveness of this combination in cancer cell lines and animal models by using the heme precursor aminolevulinic acid (ALA) as an effective enhancer of the anticancer effect of ART efficacy. In our study, we found that Artemisinin significantly inhibited CHSY1 activity, and surprisingly, we found that Artemisinin combined with anti-PD1 could co-treat CRC liver metastases. To further improve the therapeutic efficacy of Artemisinin and visualize drug metabolism in vivo, we tried PLGA-loaded Artemisinin-linked ICG probes with success. PLGA, as one of the earliest synthetic polymers developed, has high quality properties. Because of its ability to control drug release and prevent drug or gene decomposition, it has excellent application potential in the field of drug research and development. In recent years, it has also attracted attention in the diagnosis and treatment of cancer [59, 60]. ICG has an excitation peak of 780 nm and an emission peak of 820 nm, respectively. ICG is a water-soluble trichlosan dye used in near infrared imaging. Since its approval by the US Food and Drug Administration (FDA) in 1954, it has been widely used clinically as a near-infrared fluorescent moiety. ICG is often used to locate liver tumors during surgery. After 0.25 to 0.5 mg/kg ICG is administered intravenously 12 h before surgery, it is rapidly absorbed by both tumor and non-tumor liver cells. Moreover, ICG has a short half-life and can be metabolized through the biliary tract after just a few hours [61, 62]. In our study, after injection of PLGA-loaded Artemisinin coupling agent into the tail vein of mice, we observed that the drug concentration in the liver peaked at 0.5 h, while the intravenously injected drug rarely aggregated and metabolized in the liver and kidneys, suggesting the specificity of Artemisinin against CRC liver metastases. Interdisciplinary research can integrate resources, easily achieve major original scientific breakthroughs, integrate ideas, and solve more problems. Combining molecular and pharmacological with clinical translation in the experimental phase of the study will facilitate the exploration of new clinical treatment options and provide new prospects for the treatment of CRC liver metastases.

## Conclusion

**Fig. 9:**
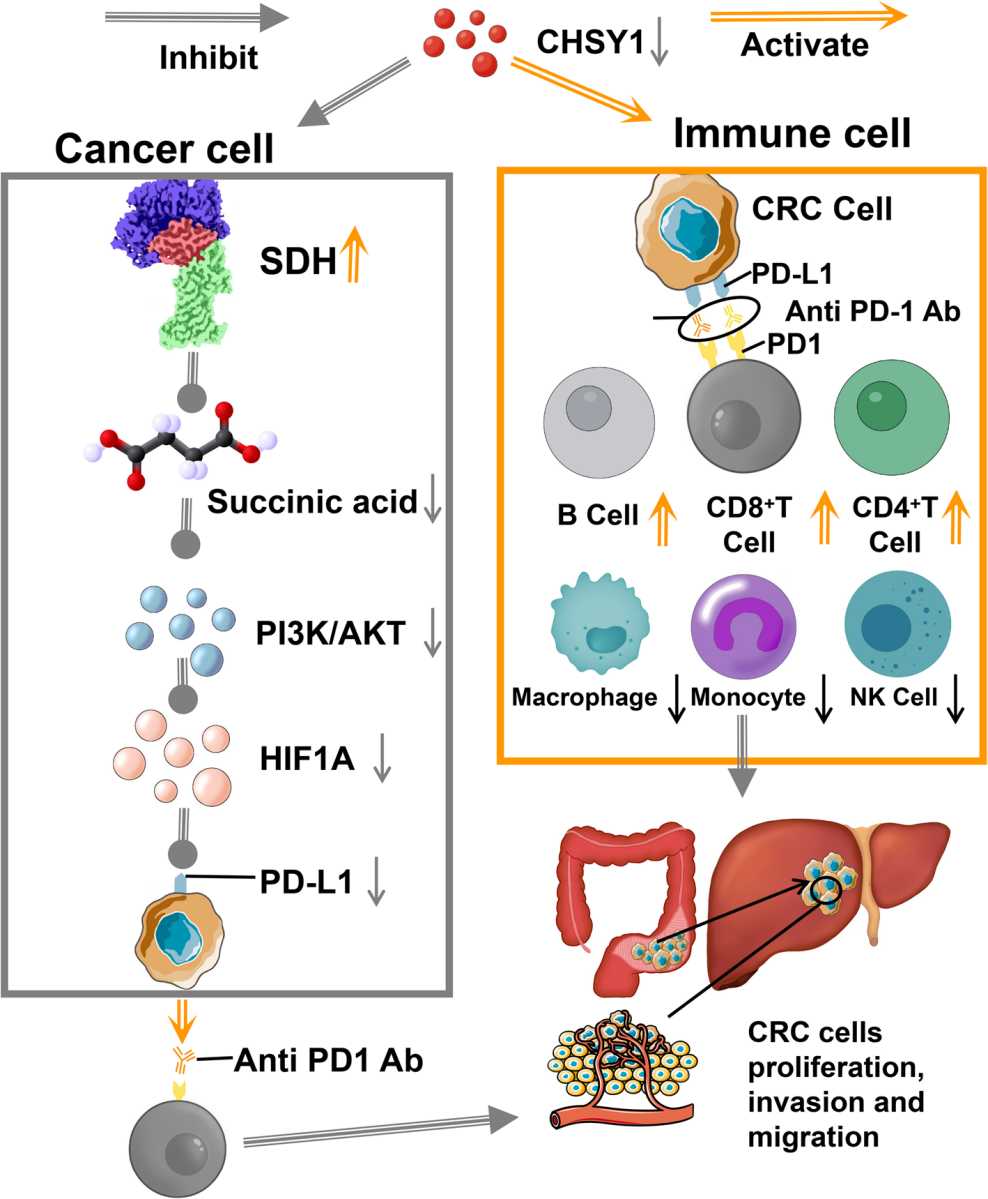
Pattern diagram showing that CHSY1 could promote CD8^+^ T cell exhaustion by activating the succinate metabolism pathway leading to CRC liver metastasis based on CRISPR/Cas9 results.The combination of CHSY1 knockdown and anti-PD1 contributed to synergistic resistance against liver metastasis in CRC. Artemisinin could significantly inhibit the activity of CHSY1, and the combination of anti-PD1 can synergistically treat CRC liver metastasis. PLGA-loaded Artemisinin-linked ICG probe further improves the therapeutic effect of Artemisinin and realizes the visualization of drug metabolism in vivo

According to CRISPR/Cas9 results, CHSY1 could promote CD8^+^ T cell exhaustion through activation of the succinate metabolic pathway, leading to CRC liver metastasis. The combination of CHSY1 knockdown and anti-PD1 contributes to synergistic resistance to CRC liver metastasis. Artemisinin significantly inhibits CHSY1 activity and in combination with anti-PD1 could synergistically treat CRC liver metastases. PLGA-loaded Artemisinin-linked ICG probes further improves the therapeutic efficacy of Artemisinin and enables visualization of drug metabolism in vivo (Fig. 9).

### Supplementary Information

**Additional file 1: Table S1.** Primer sequences, shRNAs used in this study. **Figure S1.** (A) Detailed analysis process for bioinformatic analysis after obtaining raw data of sufficient sequencing depth. (B-C) Average matrix values and average sequencing error rates of metastatic (B) and primary (C) foci at different sequencing sites were obtained. (D-E) The separation of AT and GC in metastatic foci (D)and primary foci (E) was detected by statistical analysis of alkali content distribution. **Figure S2.** (A-B) Detection of primary CRC, adjacent normal bowel tissue, CRC liver metastases, adjacent normal live, and preoperative blood samples using single-cell RNA sequencing. Taxonomic definition of specific gene markers using UMAP plots identified 12 cell clusters. (C-D) The bar chart and violin figure showed that CHSY1 was relatively highly expressed in the total sample analysis of the cancer cell population. (E)The dot plot shows the expression of CHSY in colorectal cancer primary tumor(CT), adjacent tissue(CP), liver metastases(LT), and adjacent metastatic tissue(LP). **Figure S3.** (A-B) Gene overexpression efficiency of pcDNA3-CHSY1 assessed by qRT-PCR and Western blotting. (C-D) pcDNA3-CHSY1 promoted the proliferation of HCT116 and LOVO cells according to the results of CCK-8 and EdU assays. (E-F) Transwell and wound healing assays show that pcDNA3-CHSY1 significantly promoted the invasive and migratory functions of HCT116 and LOVO cells.Scale bar, 100μm. **, *P* < 0.01; ***, *P* < 0.001;****,*P* < 0.0001. **Figure S4.** The TISIDB database was used to predict the correlation between CHSY1 expression and immune factors(for example,PD-L1, PD1, LAG3, IDO1 and CTLA4). **Figure S5.** Single, viable and intact CD45^+^ 21 immune cells were selected and circulated in liver metastases. There are a total of 33 cell clusters, each of which was defined based on markers specific to the respective. **Figure S6.** Negative patterns of metabonomics analysis of down-regulating CHSY1 in CRC cells. (A)Volcano maps classify metabolites by HMDB. (B) circos plot showing correlations between multiple differential metabolites. (C) According to the structure and function of the metabolites, the different metabolites in each control group were classified and counted, and the classification results of the substances in the KEGG and HMDB databases were provided respectively. (D) Top 20 up-regulated metabolites and top 20 down-regulated metabolites. (E-F) KEGG pathway analysis indicated the main concentrated pathways of these differential metabolites. (G) Sankey diagram visualization of data flow trends between down-regulated metabolites and various pathways. **Figure S7.** Positive patterns of metabonomics analysis of down-regulating CHSY1 in CRC cells. (A)Volcano maps classify metabolites by HMDB. (B) circos plot showing correlations between multiple differential metabolites. (C) According to the structure and function of the metabolites, the different metabolites in each control group were classified and counted, and the classification results of the substances in the KEGG and HMDB databases were provided respectively. (D) Top 20 up-regulated metabolites and top 20 down-regulated metabolites. (E-F) KEGG pathway analysis indicated the main concentrated pathways of these differential metabolites. (G) Sankey diagram visualization of data flow trends between down-regulated metabolites and various pathways. **Figure S8**. (A-B) Inhibitory effect of artemisinin on CHSY1 assessed by qRT-PCR and Western blotting. (C-D) Artemisinin inhibited the proliferation of HCT116 and LOVO cells according to the results of CCK-8 and EdU assays. (E-F) Transwell and wound healing assays show that Artemisinin significantly inhibited the invasive and migratory functions of HCT116 and LOVO cells.**, *P* < 0.01; ***, *P* < 0.001;****, *P* < 0.0001. **Figure S9.** (A-C) Three-dimensional diagram and two-dimensional diagram show artemisinin can bind to the 429 PHE amino acid residue, 410 MET amino acid residue, 295 SER amino acid residue and 409 VAL amino acid residue of the receptor protein CHSY1 through hydrophobic forces. (D-E) Immunohistochemistry results of CD8, CD4, Ki67, PD-L1 and PD1 expression in the respective groups.*, *P* < 0.05; **, *P* < 0.01; ***, *P* < 0.001;****, *P* < 0.000.

## Data Availability

All data and material are available within the article.
