# Supplementary material for: CHSY1 promotes CD8+ T cell exhaustion through activation of succinate metabolism pathway leading to colorectal cancer liver metastasis based on CRISPR/Cas9 screening
Source: J Exp Clin Cancer Res. 2023 Sep 25;42:248. doi: 10.1186/s13046-023-02803-0 (PMC10519095; doi:10.1186/s13046-023-02803-0)
Supplement: Supplementary file 1 — Additional file 1: Table S1. Primer sequences, shRNAs used in this study. Figure S1. (A) Detailed analysis process for bioinformatic analysis after obtaining raw data of sufficient sequencing depth. (B-C) Average matrix values and average sequencing error rates of metastatic (B) and primary (C) foci at different sequencing sites were obtained. (D-E) The separation of AT and GC in metastatic foci (D)and primary foci (E) was detected by statistical analysis of alkali content distribution. Figure S2. (A-B) Detection of primary CRC, adjacent normal bowel tissue, CRC liver metastases, adjacent normal live, and preoperative blood samples using single-cell RNA sequencing. Taxonomic definition of specific gene markers using UMAP plots identified 12 cell clusters. (C-D) The bar chart and violin figure showed that CHSY1 was relatively highly expressed in the total sample analysis of the cancer cell population. (E)The dot plot shows the expression of CHSY in colorectal cancer primary tumor(CT), adjacent tissue(CP), liver metastases(LT), and adjacent metastatic tissue(LP). Figure S3. (A-B) Gene overexpression efficiency of pcDNA3-CHSY1 assessed by qRT-PCR and Western blotting. (C-D) pcDNA3-CHSY1 promoted the proliferation of HCT116 and LOVO cells according to the results of CCK-8 and EdU assays. (E-F) Transwell and wound healing assays show that pcDNA3-CHSY1 significantly promoted the invasive and migratory functions of HCT116 and LOVO cells.Scale bar, 100μm. **, P < 0.01; ***, P < 0.001;****,P < 0.0001. Figure S4. The TISIDB database was used to predict the correlation between CHSY1 expression and immune factors(for example,PD-L1, PD1, LAG3, IDO1 and CTLA4). Figure S5. Single, viable and intact CD45+ 21 immune cells were selected and circulated in liver metastases. There are a total of 33 cell clusters, each of which was defined based on markers specific to the respective. Figure S6. Negative patterns of metabonomics analysis of down-regulating CHSY1 in CRC cells. (A)Volcano m [file 13046_2023_2803_MOESM1_ESM.zip › renamed_fc0d4.pdf]

Supplementary figure legends:

Figure S1: (A)Detailed analysis process for bioinformatic analysis after obtaining raw data of sufficient sequencing depth. (B-C) Average matrix values and average sequencing error rates of metastatic(D) and primary(E) foci at different sequencing sites were obtained. (D-E) The separation of AT and GC in metastatic foci (F)and primary foci (G)was detected by statistical analysis of alkali content distribution.

Figure S2: (A-B) Detection of primary CRC, adjacent normal bowel tissue, CRC liver metastases, adjacent normal liver tissue, and preoperative blood samples using single-cell RNA sequencing. Taxonomic definition of specific gene markers using UMAP plots identified 12 cell clusters. (C-D) The bar chart and violin figure showed that CHSY1 was relatively highly expressed in the total sample analysis of the cancer cell population. (E)The dot plot shows the expression of CHSY in colorectal cancer primary tumor(CT), adjacent tissue(CP), liver metastases(LT), and adjacent metastatic tissue(LP).

Figure S3: (A-B) Gene overexpression efficiency of pcDNA3-CHSY1 assessed by qRT-PCR and Western blotting. (C-D) pcDNA3-CHSY1 promoted the proliferation of HCT116 and LOVO cells according to the results of CCK-8 and EdU assays. (E-F) Transwell and wound healing assays show that pcDNA3-CHSY1 significantly promoted the invasive and migratory functions of HCT116 and LOVO cells.Scale bar, 100μm. **, $P < 0.01$; ***, $P < 0.001$;****, $P < 0.0001$.

Figure S4: The TISIDB database was used to predict the correlation between CHSY1 expression and immune factors(for example,PD-L1, PD1, LAG3, IDO1 and CTLA4).

Figure S5: Single, viable and intact $CD45^+$ immune cells were selected and circulated in liver metastases. There are a total of 33 cell clusters, each of which was defined based on markers specific to the respective cell type.

Figure S6: Negative patterns of metabonomics analysis of down-regulating CHSY1 in CRC cells. (A)Volcano maps classify metabolites by HMDB. (B) circos plot showing correlations between multiple differential metabolites. (C) According to the structure and function of the metabolites, the different metabolites in each control group were classified and counted, and the classification results of the substances in the KEGG and HMDB databases were provided respectively. (D) Top 20 up-regulated metabolites and top 20 down-regulated metabolites. (E-F) KEGG pathway analysis indicated the main concentrated pathways of these differential metabolites. (G) Sankey diagram visualization of data flow trends between down-regulated metabolites and various pathways.

Figure S7: Positive patterns of metabonomics analysis of down-regulating CHSY1 in CRC cells. (A)Volcano maps classify metabolites by HMDB. (B) circos plot showing correlations between multiple differential metabolites. (C) According to the structure and function of the metabolites, the different metabolites in each control group were classified and counted, and the classification results of the substances in the KEGG and HMDB databases were provided respectively. (D) Top 20 up-regulated metabolites and top 20 down-regulated metabolites. (E-F) KEGG pathway analysis indicated the main concentrated pathways of these differential metabolites. (G) Sankey diagram visualization of data flow trends between down-regulated metabolites and various pathways.

Figure S8: (A-B) Inhibitory effect of artemisinin on CHSY1 assessed by qRT-PCR and Western blotting. (C-D) Artemisinin inhibited the proliferation of HCT116 and LOVO cells according to the results of CCK-8 and EdU assays. (E-F) Transwell and wound healing assays show that Artemisinin significantly inhibited the invasive and migratory functions of HCT116 and LOVO cells.**, $P < 0.01$; ***, $P < 0.001$;****, $P < 0.0001$.

Figure S9: (A-C) Three-dimensional diagram and two-dimensional diagram show artemisinin can bind to the 429 PHE amino acid residue, 410 MET amino acid residue, 295 SER amino acid residue and 409 VAL amino acid residue of the receptor protein CHSY1

through hydrophobic forces. (D-E) Immunohistochemistry results of CD8, CD4, Ki67, PD-L1 and PD1 expression in the respective groups.*, $P < 0.05$; **, $P < 0.01$; ***, $P < 0.001$;****, $P < 0.0001$.

Table S1. **Primer sequences, shRNAs used in this study.**

Primer/shRNA/siRNA		Sequence (5'-3')
	Forward primer	CTGGGCACCACGGAAGAAAT
human-CHSY1	Reverse primer	GGCACCATTCTCCGAAGCA
	Forward primer	TGGCATTTGCTGAACGCATTT
human-PD-L1	Reverse primer	TGCAGCCAGGTCTAATTGTTTT
	Forward primer	GTGGCCGCCTACAGAACAT
Mouse-CHSY1	Reverse primer	TCTGCCCTCATAAACCACTCG
	Forward primer	AGGCCGGTGCTGAGTATGTC
mouse-GAPDH	Reverse primer	TGCCTGCTTCACCACCTTCT
human-sh1-CHSY1	ccggccCAGTTTAACAATGAATCTTctcgagAAGATTCATTGTTAAACTGggtttttg	
human-sh2-CHSY1	ccggccAAGAGAATCAATCAGGAATctcgagATTCCTGATTGATTCTCTTggtttttg	
human-sh3-CHSY1	ccggcgACATTGTCATGCAGGTCATctcgagATGACCTGCATGACAATGTcgtttttg	
mouse-sh1-CHSY1	ccggctGTCGTCTGTGACCCTAATCctcgagGATTAGGGTCACAGACGACagtttttg	
mouse-sh2-CHSY1	ccggagAACAAGAAAGGGTACATTActcgagTAATGTACCCTTTCTTGTTcttttttg	
mouse-sh3-CHSY1	ccggtgGCAAGTGTCTCCGGGAAATctcgagATTTCCCGGAGACACTTGCcatttttg	
