# Supplementary figures and images for: CHSY1 promotes CD8+ T cell exhaustion through activation of succinate metabolism pathway leading to colorectal cancer liver metastasis based on CRISPR/Cas9 screening
Source: J Exp Clin Cancer Res. 2023 Sep 25;42:248. doi: 10.1186/s13046-023-02803-0 (PMC10519095; doi:10.1186/s13046-023-02803-0)

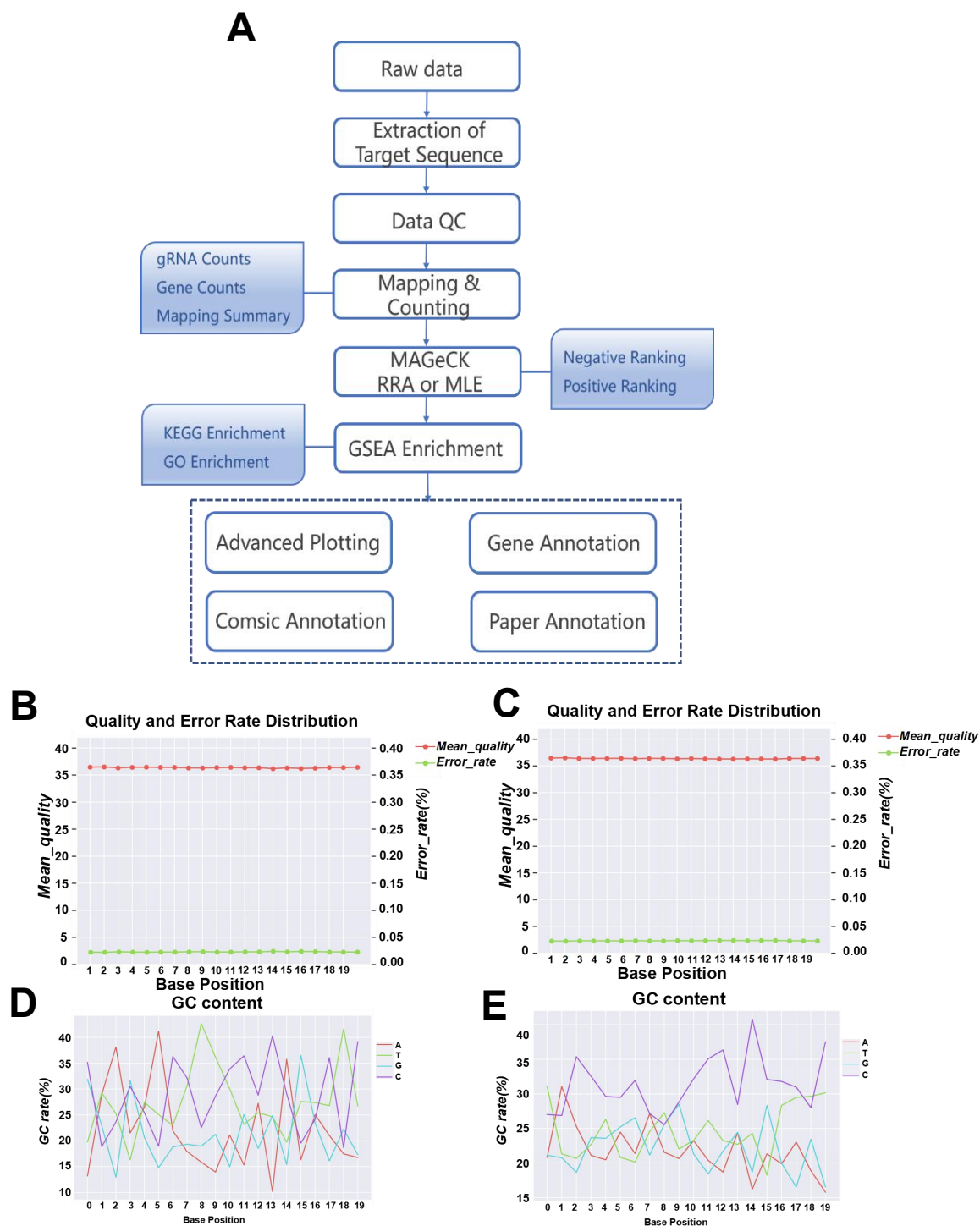

Figure S2

A

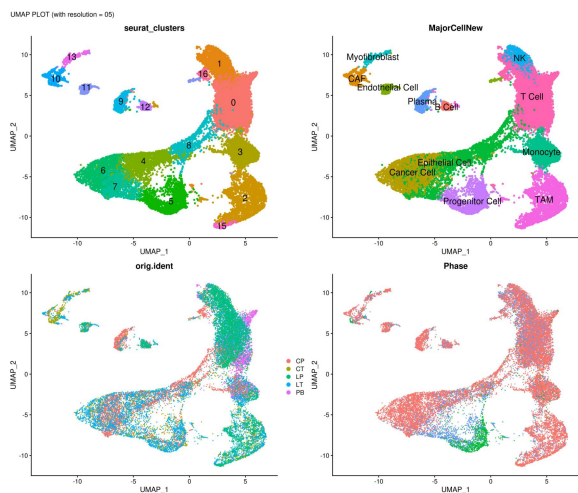

C

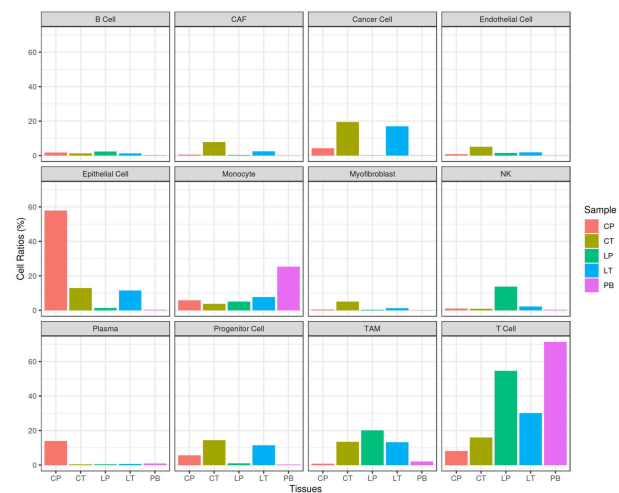

B

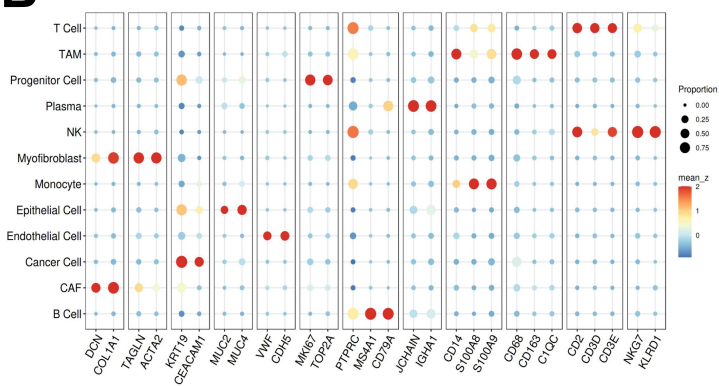

D

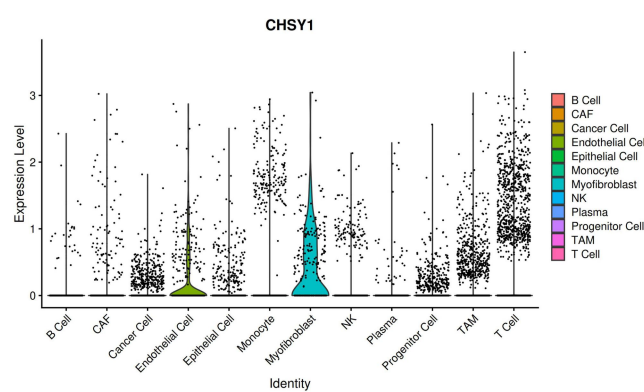

E

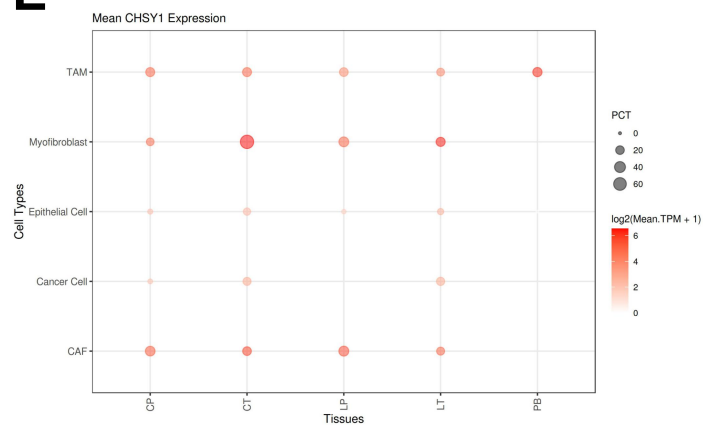

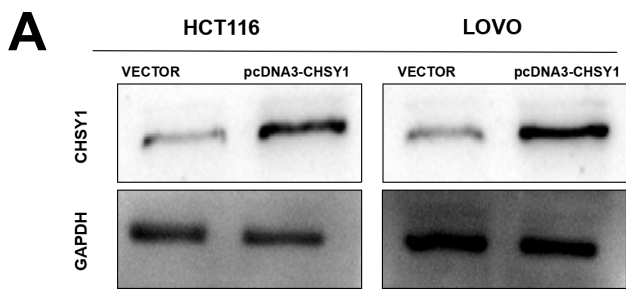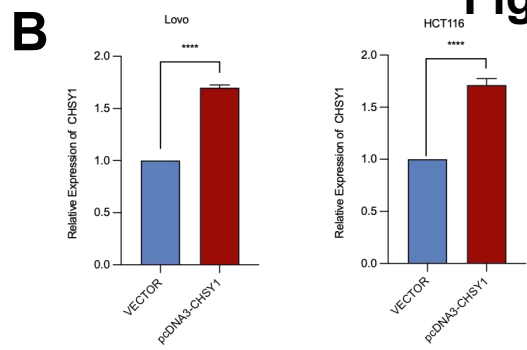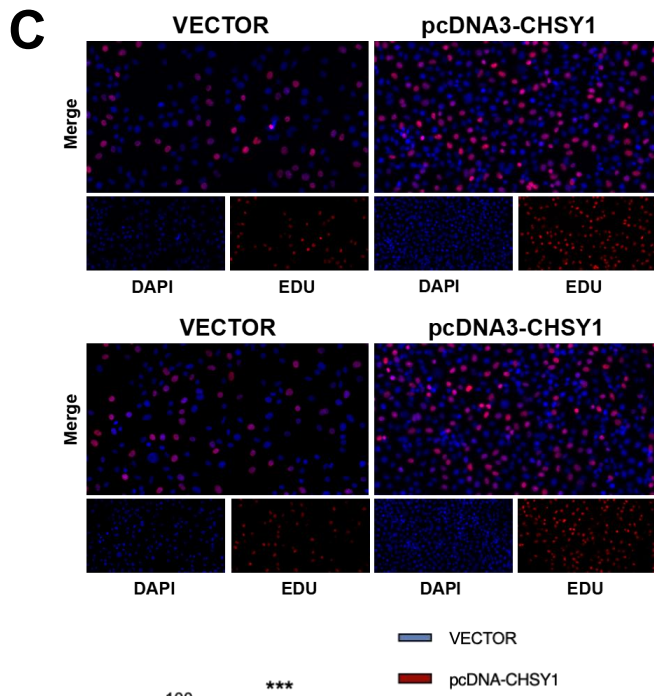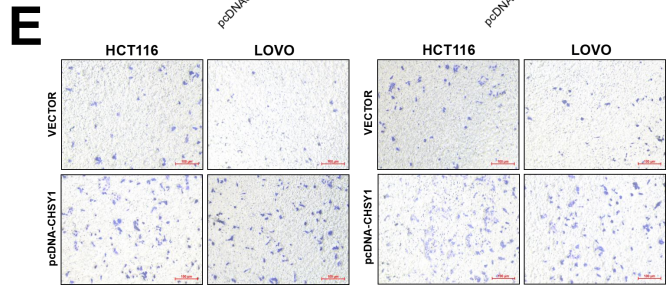

Migration

Invasion

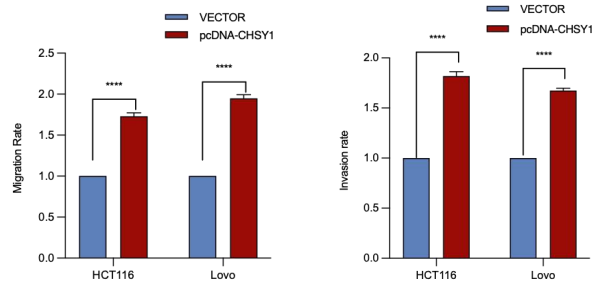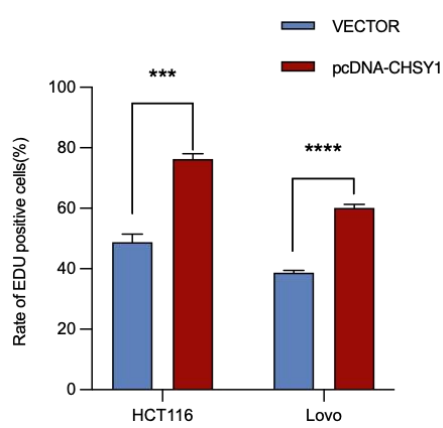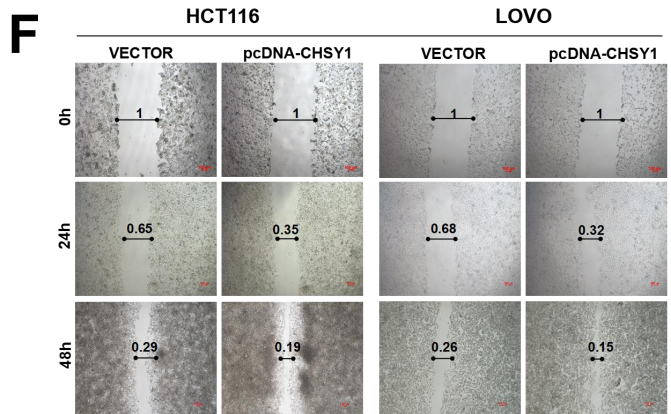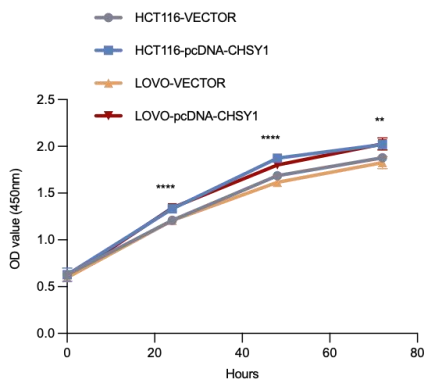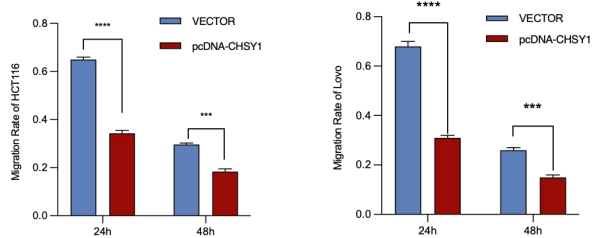

Figure S4

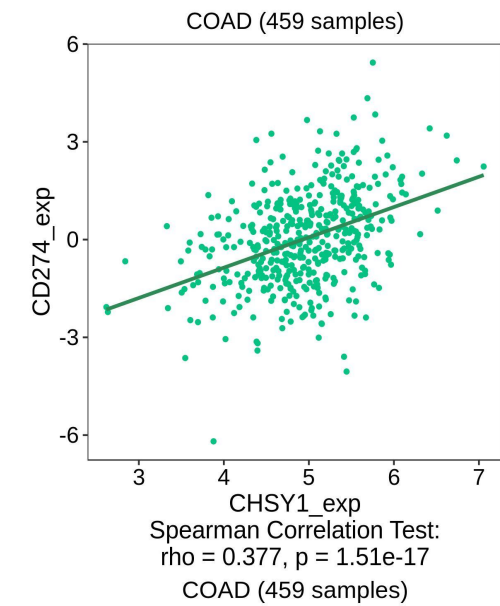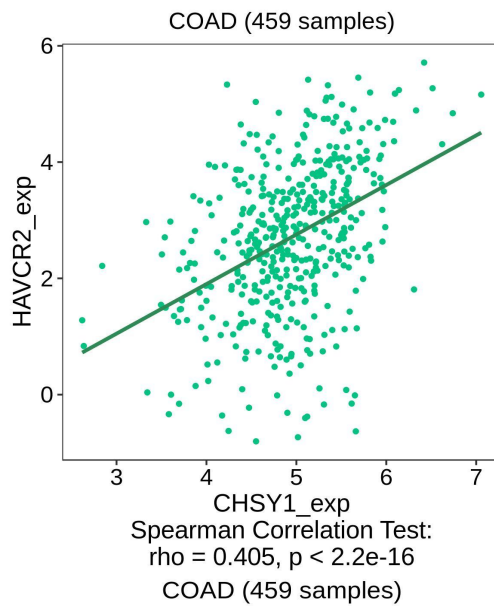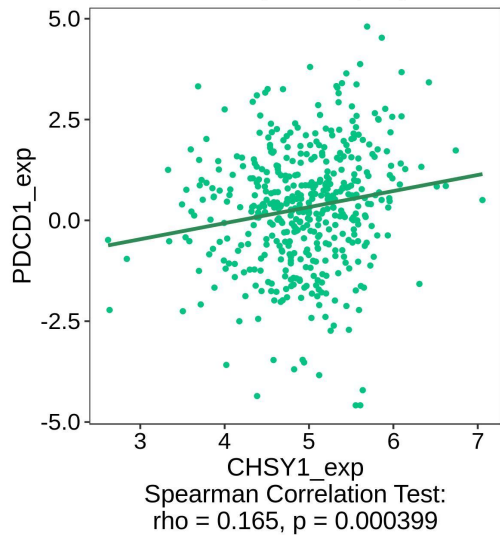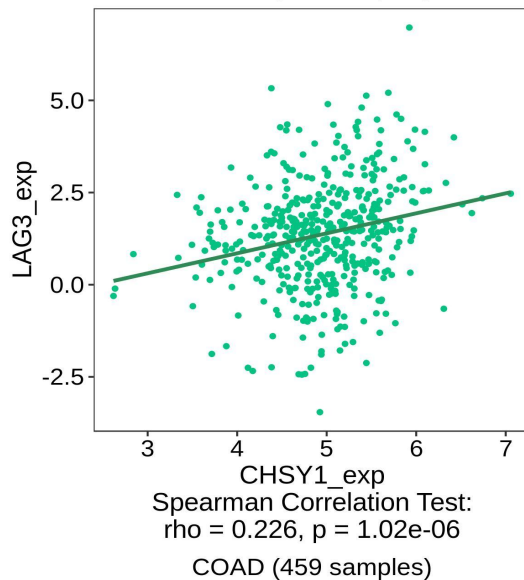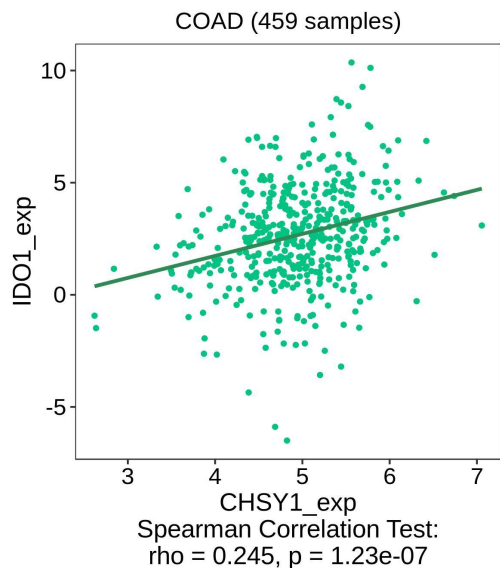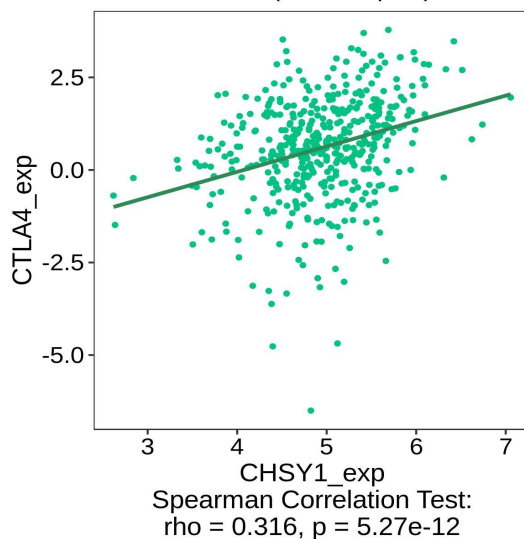

# Figure S5

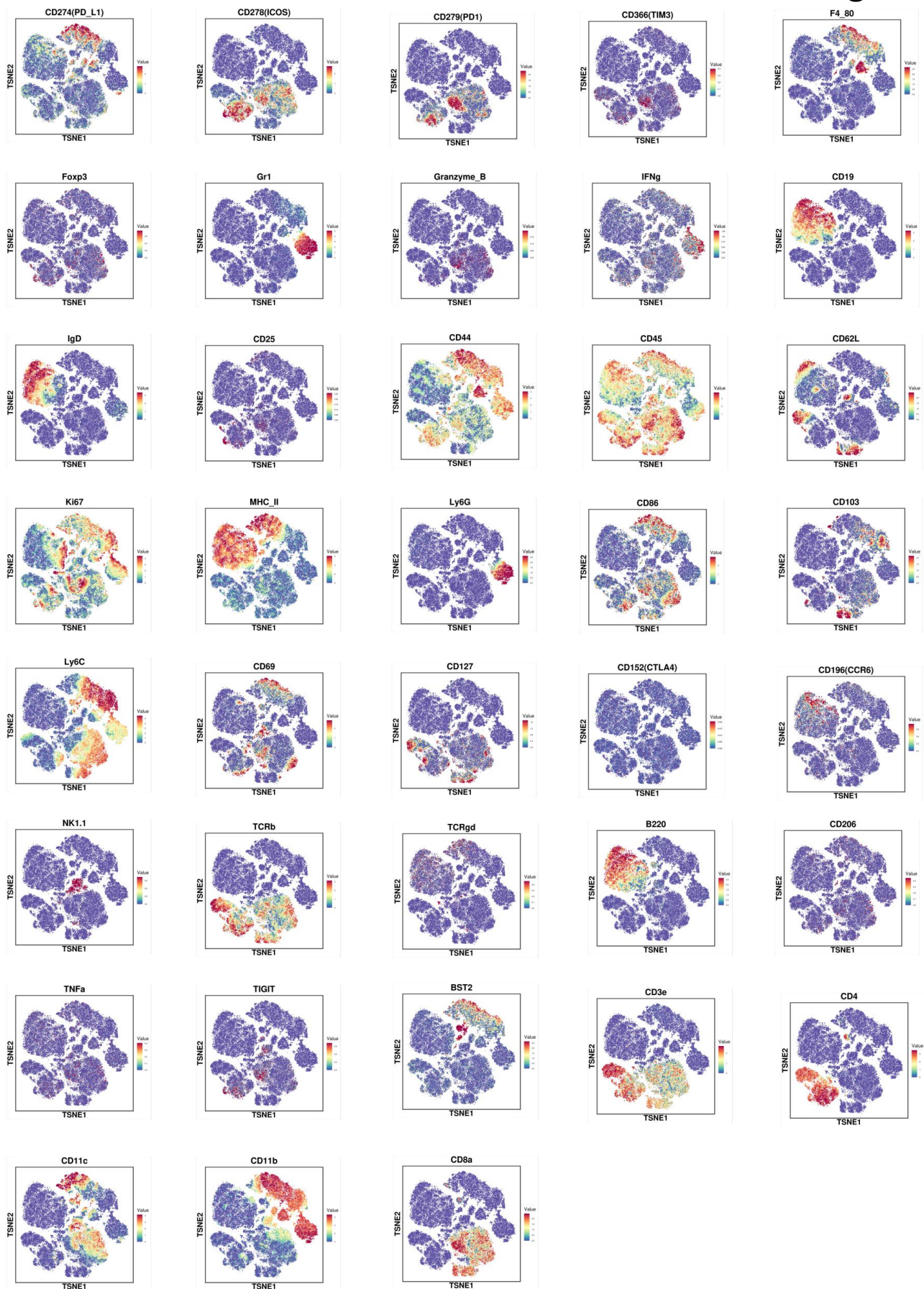

# B

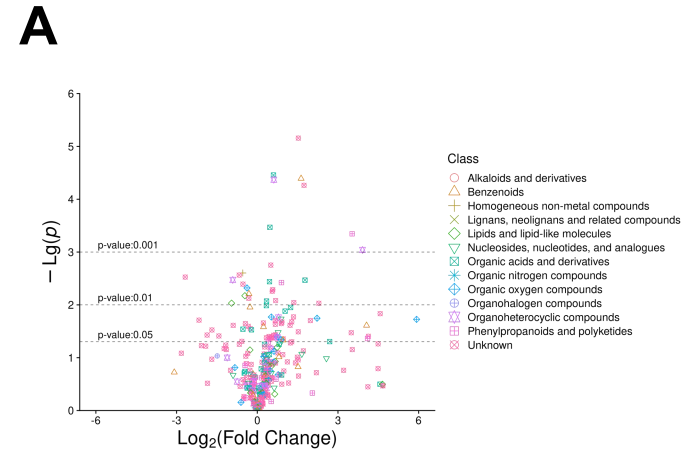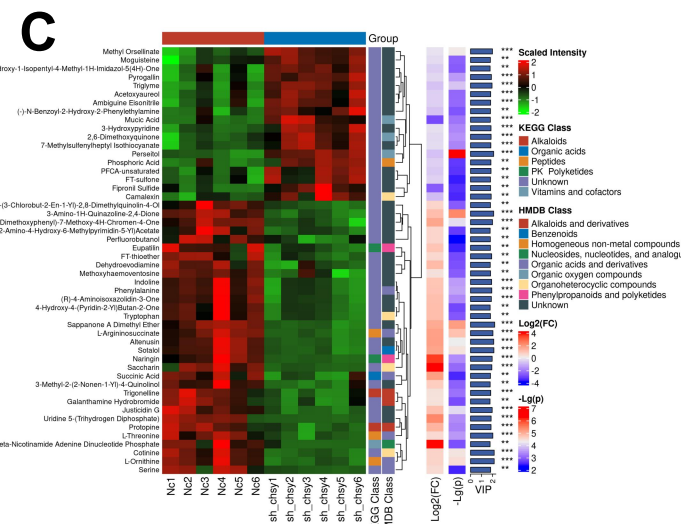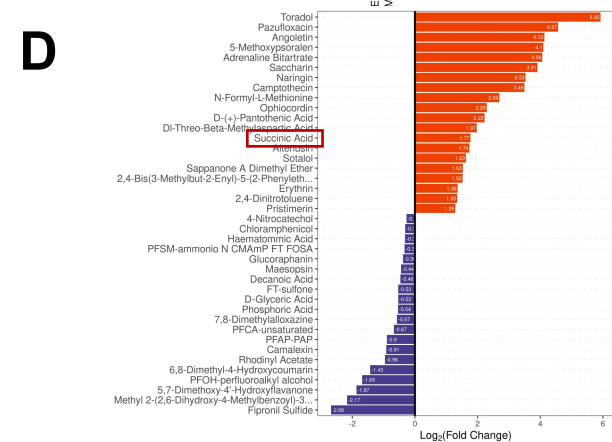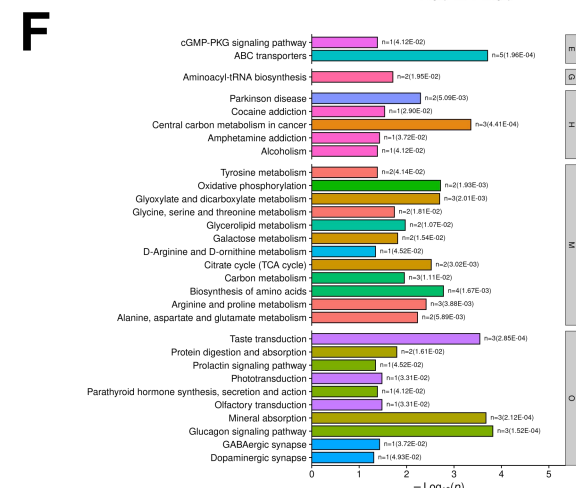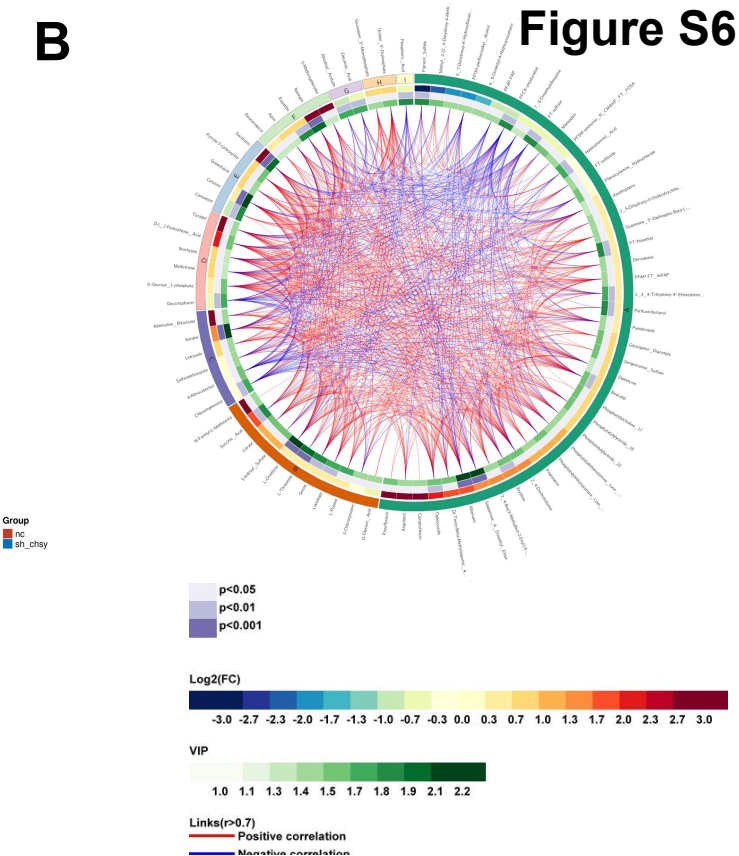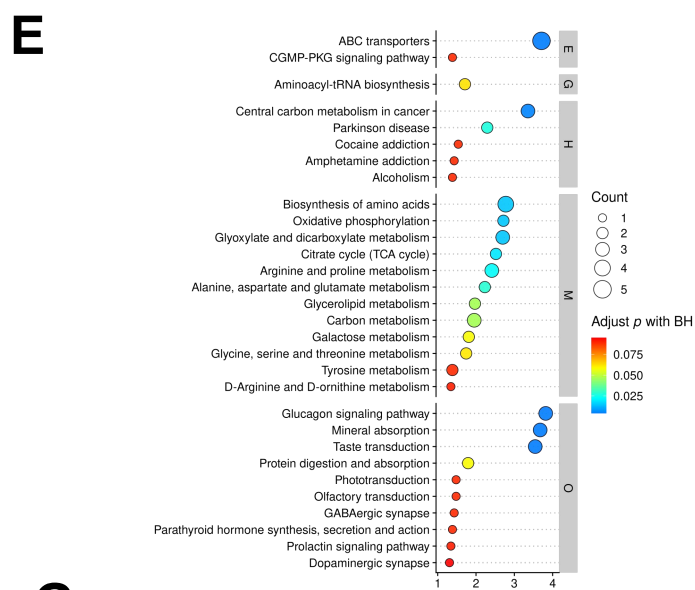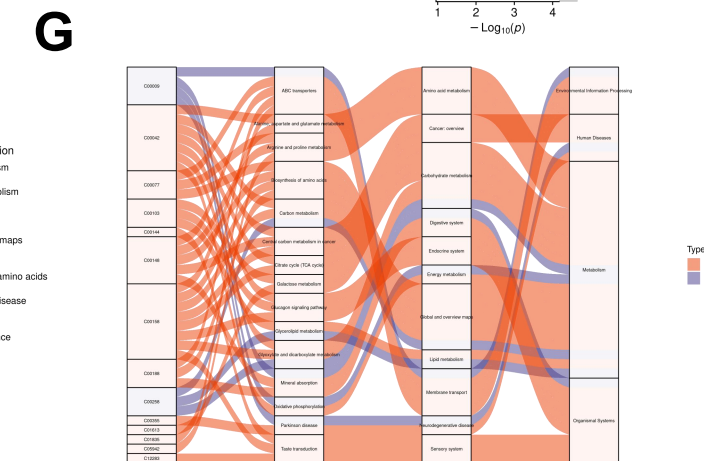

Figure S7

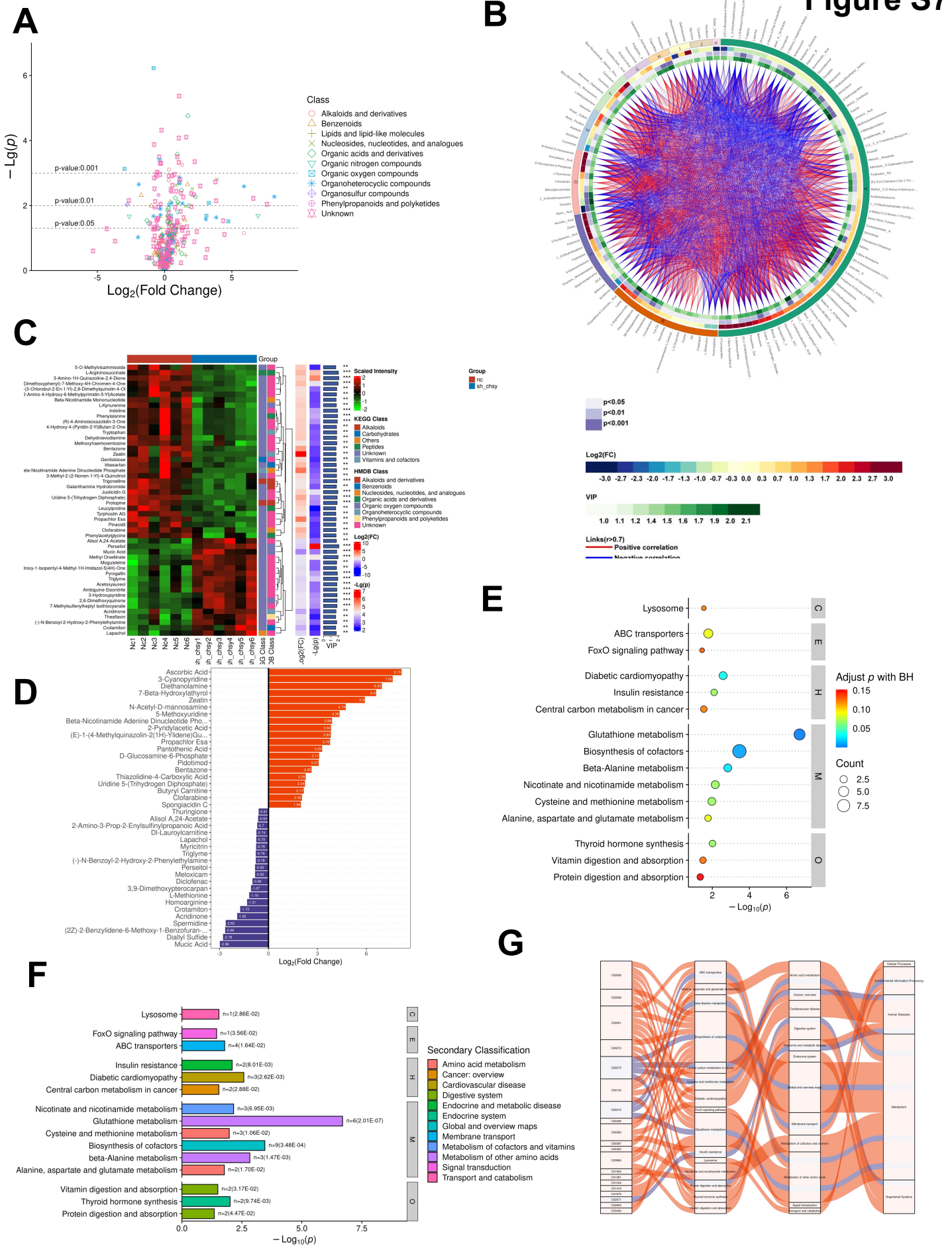

**Figure S8**

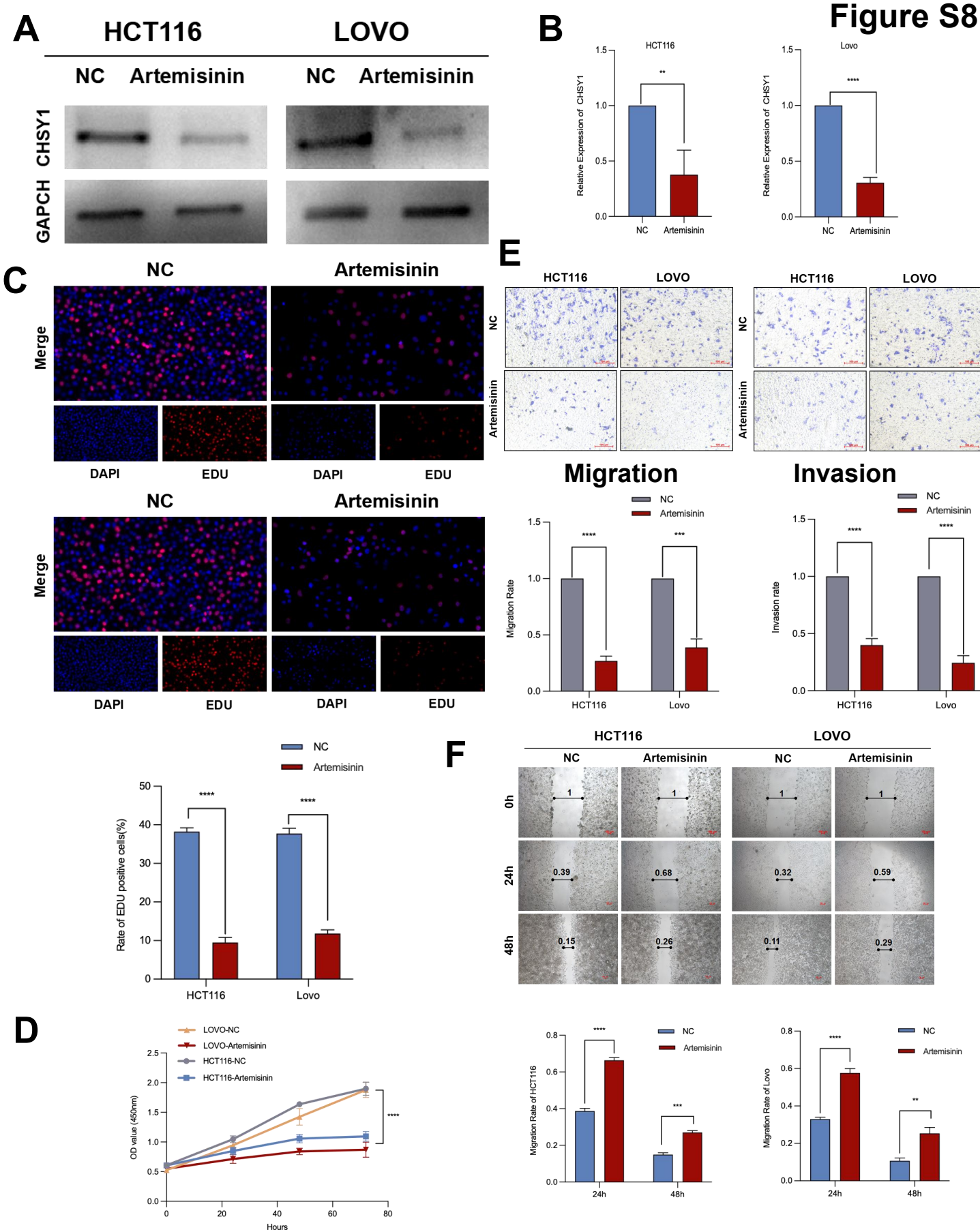

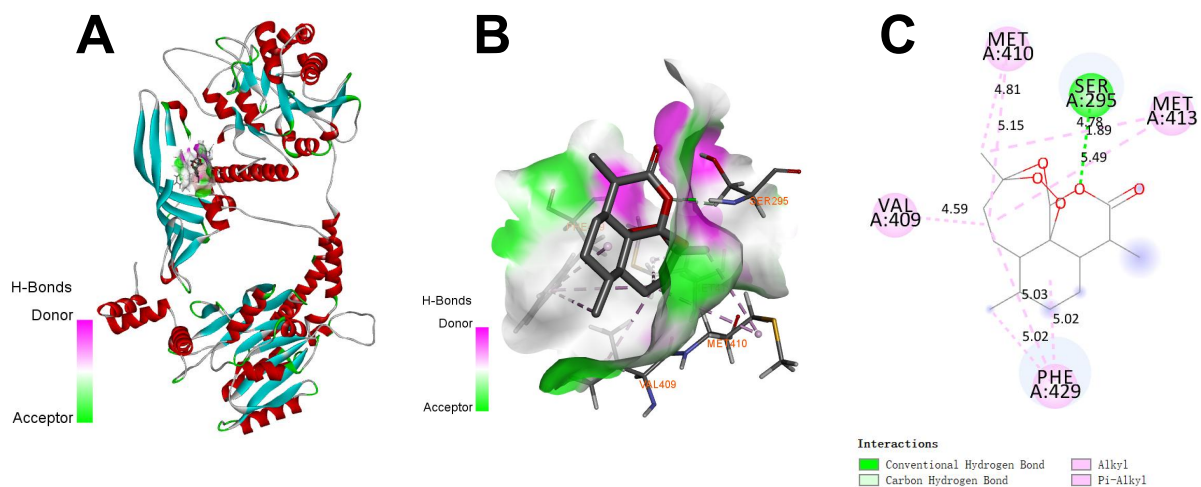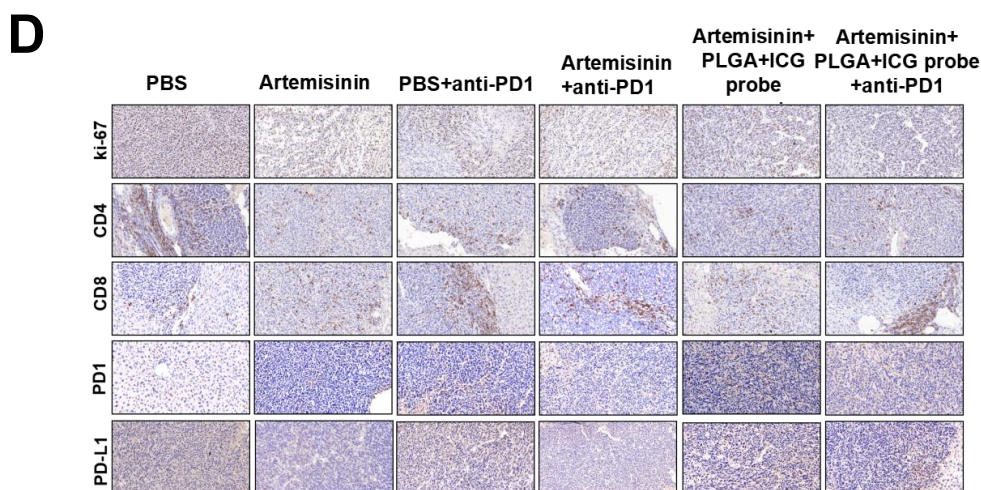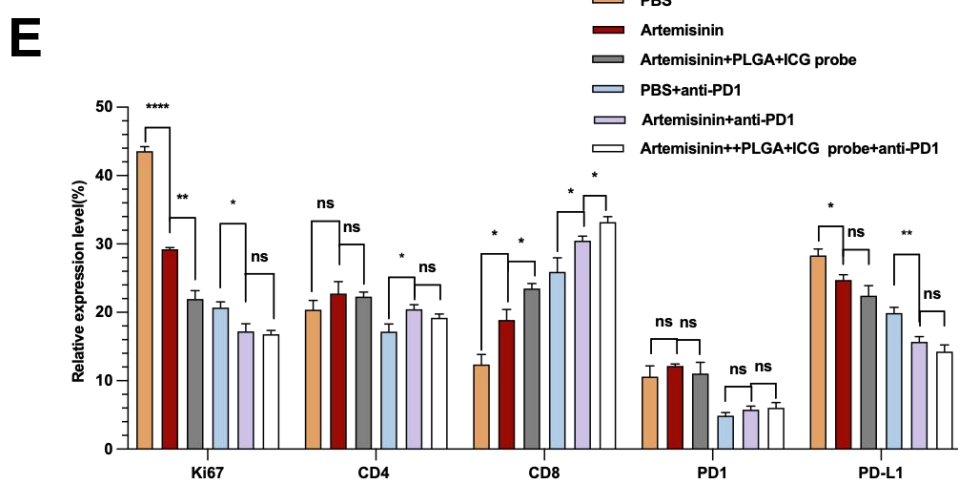

Supplement: Supplementary file 1 — Additional file 1: Table S1. Primer sequences, shRNAs used in this study. Figure S1. (A) Detailed analysis process for bioinformatic analysis after obtaining raw data of sufficient sequencing depth. (B-C) Average matrix values and average sequencing error rates of metastatic (B) and primary (C) foci at different sequencing sites were obtained. (D-E) The separation of AT and GC in metastatic foci (D)and primary foci (E) was detected by statistical analysis of alkali content distribution. Figure S2. (A-B) Detection of primary CRC, adjacent normal bowel tissue, CRC liver metastases, adjacent normal live, and preoperative blood samples using single-cell RNA sequencing. Taxonomic definition of specific gene markers using UMAP plots identified 12 cell clusters. (C-D) The bar chart and violin figure showed that CHSY1 was relatively highly expressed in the total sample analysis of the cancer cell population. (E)The dot plot shows the expression of CHSY in colorectal cancer primary tumor(CT), adjacent tissue(CP), liver metastases(LT), and adjacent metastatic tissue(LP). Figure S3. (A-B) Gene overexpression efficiency of pcDNA3-CHSY1 assessed by qRT-PCR and Western blotting. (C-D) pcDNA3-CHSY1 promoted the proliferation of HCT116 and LOVO cells according to the results of CCK-8 and EdU assays. (E-F) Transwell and wound healing assays show that pcDNA3-CHSY1 significantly promoted the invasive and migratory functions of HCT116 and LOVO cells.Scale bar, 100μm. **, P < 0.01; ***, P < 0.001;****,P < 0.0001. Figure S4. The TISIDB database was used to predict the correlation between CHSY1 expression and immune factors(for example,PD-L1, PD1, LAG3, IDO1 and CTLA4). Figure S5. Single, viable and intact CD45+ 21 immune cells were selected and circulated in liver metastases. There are a total of 33 cell clusters, each of which was defined based on markers specific to the respective. Figure S6. Negative patterns of metabonomics analysis of down-regulating CHSY1 in CRC cells. (A)Volcano m [file 13046_2023_2803_MOESM1_ESM.zip › renamed_6a072.pdf]
